# Rifaximin-α for liver fibrosis in patients with alcohol-related liver disease (GALA-RIF): a randomised, double-blind, placebo-controlled, phase 2 trial

**DOI:** 10.1016/S2468-1253(23)00010-9

**Published:** 2023-03-06

**Authors:** Mads Israelsen, Bjørn Stæhr Madsen, Nikolaj Torp, Stine Johansen, Camilla Dalby Hansen, Sönke Detlefsen, Peter Andersen, Johanne Kragh Hansen, Katrine Prier Lindvig, Ditlev Nytoft Rasmussen, Katrine Holtz Thorhauge, Maria Kjærgaard, Morten Karsdal, Torben Hansen, Manimozhiyan Arumugam, Jonel Trebicka, Maja Thiele, Aleksander Krag, GALAXY, Ema Anastasiadou, Manimozhian Arumugam, Peer Bork, Torben Hansen, Roland Henrar, Hans Israelsen, Morten Karsdal, Cristina Legido-Quigley, Hans Olav Melberg, Maja Thiele, Jonel Trebicka, Aleksander Krag, MicrobLiver Consortia, Peer Bork, Mathias Mann, Jelle Matthijnssens, Aleksander Krag, Torben Hansen

**Affiliations:** a Odense Liver Research Center, Department of Gastroenterology and Hepatology, Odense University Hospital, Odense, Denmark; b Department of Pathology, Odense University Hospital, Odense, Denmark; c Institute of Clinical Research, Faculty of Health Sciences, University of Southern Denmark, Odense, Denmark; d Department of Molecular Medicine, Faculty of Health Sciences, University of Southern Denmark, Odense, Denmark; e Nordic Bioscience Biomarkers and Research, Herlev, Denmark; f Novo Nordisk Foundation Center for Basic Metabolic Research, University of Copenhagen, Copenhagen, Denmark; g Department of Internal Medicine B, Münster University Hospital, WWU, Münster, Germany; h European Foundation for Study of Chronic Liver Failure, Barcelona, Spain

## Abstract

**Background:**

Alcohol is the leading cause of liver-related mortality worldwide. The gut–liver axis is considered a key driver in alcohol-related liver disease. Rifaximin-α improves gut-barrier function and reduces systemic inflammation in patients with cirrhosis. We aimed to compare the efficacy and safety of rifaximin-α with placebo in patients with alcohol-related liver disease.

**Methods:**

GALA-RIF was an investigator-initiated, randomised, double-blind, placebo-controlled, single-centre, phase 2 trial done at Odense University Hospital in Denmark. Eligible participants were adults (aged 18–75 years) who had current or previous alcohol overuse (at least 1 year with ≥24 g of alcohol per day for women and ≥36 g of alcohol per day for men), biopsy-proven alcohol-related liver disease, and no previous hepatic decompensation. Patients were randomly allocated (1:1) through a web-based randomisation system to receive oral rifaximin-α (550 mg) twice daily or matched placebo for 18 months. Randomisation was done in blocks of four and stratified according to fibrosis stage and alcohol abstinence. Participants, sponsor, investigators, and nurses involved in the study were masked to the randomisation outcome. The primary endpoint was a histological decrease from baseline to 18-month treatment of at least one fibrosis stage, according to the Kleiner fibrosis score. We also assessed the number of patients with progression by at least one fibrosis stage from baseline to 18 months. Primary analyses were done in the per-protocol and modified intention-to-treat populations; safety was assessed in the full intention-to-treat population. The per-protocol population was defined as all randomly assigned patients who did not present serious protocol violations, who ingested at least 75% of the treatment, and who were not withdrawn from the study due to non-adherence (interruption of treatment for 4 weeks or more). Participants receiving at least one dose of the intervention were included in the modified intention-to-treat analyses. This completed trial is registered with EudraCT, number 2014–001856-51.

**Findings:**

Between March 23, 2015, and Nov 10, 2021, we screened 1886 consecutive patients with a history of excessive alcohol consumption and no previous hepatic decompensation, of whom 136 were randomly assigned to either rifaximin-α (n=68) or placebo (n=68). All patients were White (100%), 114 (84%) were men, and 22 (16%) were women. 133 (98%) patients received at least one dose of the intervention and were included in the modified intention-to-treat analysis; 108 (79%) completed the trial per protocol. In the per-protocol analysis, 14 (26%) of 54 patients in the rifaximin-α group and 15 (28%) of 54 patients in the placebo group had a decrease in fibrosis stage after 18 months (odds ratio 1·10 [95% CI 0·45–2·68]; p=0·83). In the modified intention-to-treat analysis, 15 (22%) of 67 patients in the rifaximin-α group and 15 (23%) of 66 patients in the placebo group had a decrease in fibrosis stage at 18 months (1·05 [0·45–2·44]; p=0·91). In the per-protocol analysis, increase in fibrosis stage occurred in 13 (24%) patients in the rifaximin-α group and 23 (43%) patients in the placebo group (0·42 [0·18–0·98]; p=0·044). In the modified intention-to-treat analysis, increase in fibrosis stage occurred in 13 (19%) patients in the rifaximin-α group and 23 (35%) patients in the placebo group (0·45 [0·20–1·02]; p=0·055). The number of patients with adverse events (48 [71%] of 68 patients in the rifaximin-α group; 53 [78%] of 68 in the placebo group) and serious adverse events (14 [21%] in the rifaximin-α group; 12 [18%] in the placebo group) was similar between the groups. No serious adverse events were deemed related to treatment. Three patients died during the trial, but none of the deaths were considered treatment related.

**Interpretation:**

In patients with alcohol-related liver disease, rifaximin-α might reduce progression of liver fibrosis. These findings warrant confirmation in a multicentre phase 3 trial.

**Funding:**

The EU Horizon 2020 Research and Innovation Program and The Novo Nordisk Foundation.

**Research in context**

**Evidence before this study**

We searched Medline for full papers published in any language in peer-reviewed journals up to Sep 1, 2022, with the term “rifaximin” and filtered by “clinical trial” and identified 138 papers. We manually reviewed the files and identified 37 papers reporting results of randomised controlled trials of rifaximin in patients with liver disease. Among these, 35 trials studied the effect of rifaximin in preventing or treating complications of cirrhosis, one study included patients with alcohol-related hepatitis, and one study included patients with non-alcoholic fatty liver disease. Most studies used doses around 600 mg twice daily in treatment periods up to 6 months and reported that rifaximin is effective and safe to prevent hepatic encephalopathy. These results are supported by several meta-analyses. However, no studies evaluated the efficacy of rifaximin-α in liver fibrosis and its safety in patients with alcohol-related liver disease.

**Added value of this study**

GALA-RIF, an 18-month investigator-initiated, randomised, double-blind, placebo-controlled trial, showed that rifaximin-α did not lead to regression of liver fibrosis in patients with alcohol-related liver disease. However, rifaximin-α appears to reduce progression of liver fibrosis with a number needed to treat of six. As a possible explanation of this beneficial effect, rifaximin-α also reduced hepatic inflammation. Furthermore, long-term treatment with rifaximin-α was well tolerated and we observed no cases of infection with multidrug resistant bacteria or *Clostridioides difficile*.

**Implications of all the available evidence**

Our findings showing that rifaximin-α seems to reduce progression of liver fibrosis represent a major clinical breakthrough in the management of alcohol-related liver disease. The EASL–*Lancet* commission calls for actions towards early and reversible stages of liver disease. Rifaximin-α might be the first potential drug for the many patients with alcohol-related liver disease who cannot achieve long-term alcohol abstinence.

## Introduction

Alcohol is one of the leading causes of liver cirrhosis and the dominant cause of liver-related mortality worldwide.^1^, ^2^ Liver fibrosis, the precursor of cirrhosis, is the most important predictor of liver-related complications in patients with alcohol-related liver disease.^3^ Earlier identification of liver fibrosis by increased access to non-invasive tests has facilitated a rapidly growing need for therapeutics to prevent progression to cirrhosis.^4^ Around 30% of patients with moderate liver fibrosis who continue to drink alcohol will develop liver-related complications within 5 years from diagnosis.^5^ The only available treatment for liver fibrosis in patients with alcohol-related liver disease is alcohol rehabilitation,^6^ but success rates range from 20% to 60% and episodic relapse is common.^7^ Therefore, additional treatment of liver fibrosis is needed to improve the prognosis of patients with alcohol-related liver disease who cannot achieve alcohol abstinence.

The gut–liver axis is considered central for the progression of alcohol-related liver disease. Translocation of bacterial products from the gut to the liver via the portal vein might induce hepatic inflammation, and thereby fuel liver fibrogenesis and progression of fibrosis.^8^ The association between liver fibrosis severity, microbial dysbiosis, and impaired gut-barrier function supports this hypothesis.^9^ Consequently, modulation of the gut microbiome and restoration of the gut barrier are of increasing interest as a potential treatment target of liver fibrosis in patients with alcohol-related liver disease.^10^, ^11^

Antibiotics are widely used in patients with decompensated cirrhosis. They reduce complications by preventing bacterial translocation from the gut to the circulation by modulating the gut microbiome.^12^ Rifaximin-α is a broad-spectrum, bactericidal, non-absorbable derivative of rifampicin used to treat recurrent hepatic encephalopathy.^13^ The mechanism of rifaximin-α has been associated with modulating the gut microbiome and promoting gut-barrier repair in patients with decompensated cirrhosis.^14^, ^15^ However, the efficacy of rifaximin-α for the attenuation of fibrogenesis and its safety in patients with alcohol-related liver disease remain unknown.

We therefore aimed to investigate the efficacy and safety of 18 months of treatment with rifaximin-α on liver fibrosis in patients with biopsy-confirmed alcohol-related liver disease.^16^

## Methods

### Study design and participants

GALA-RIF was an investigator-initiated, randomised, double-blind, placebo-controlled, single-centre, phase 2 trial investigating the efficacy of rifaximin-α in patients with liver biopsy-proven alcohol-related liver disease. All participants were recruited at the Department of Gastroenterology and Hepatology at Odense University Hospital (Odense, Denmark).

The regional ethics committee approved the study (S-20140078) and all patients provided written informed consent. The trial was conducted according to the principles of the International Conference on Harmonization Good Clinical Practice guidelines and externally monitored by the Good Clinical Practice Unit at Odense University Hospital. A full version of the protocol is available in the appendix (pp 18–44) and a protocol paper has been published previously.^16^

We identified potential participants from a clinical study that screened for alcohol-related liver fibrosis in individuals from primary and secondary care who had previous or current alcohol overuse defined as 24 g per day or more for at least 1 year in women, and 36 g or more per day for at least 1 year for men.^17^ Sex was identified through medical records.^17^ Patients with a history of hepatic decompensation or any known liver disease were excluded. On the basis of the screening result, patients at risk of having liver fibrosis had a liver biopsy. Based on the histological assessment of liver biopsy samples, we included patients aged 18–75 years with liver fibrosis and histological features in-keeping with alcohol-related liver disease.

In the protocol, patients were required to have an Ishak score of 1–4 for liver fibrosis on biopsies.^18^ However, in an amendment of the study protocol, we replaced the Ishak score with the Kleiner score for fibrosis (F1–F4).^19^ The reason for the revision was that no accepted fibrosis grading system for alcohol-related liver disease existed at the time of the study initiation. Since then, European guidelines on the management of alcohol-related liver disease proposed non-alcoholic fatty liver disease (NAFLD) scoring systems as alternatives for fibrosis staging due to the large histological overlap between alcohol-related liver disease and NAFLD.^20^ For participant inclusion, we used the histological assessment by the on-call pathologist at the hospital. Women of child-bearing potential were required to be using a safe contraceptive and provide a negative pregnancy test. The exclusion criteria were a known allergy to rifaximin, investigator judgment that the patient would not be compliant with trial medicine, antibiotic treatment in the previous 4 weeks, contraindications for liver biopsy, cancer or other debilitating disease with a life expectancy of less than 1 year, concurrent liver disease other than alcohol-related disease, HIV, severe alcohol-related hepatitis, and not being able to speak or read Danish.

### Randomisation and masking

Participants were randomly allocated (1:1) to receive rifaximin-α or placebo. The hospital pharmacy at Odense University Hospital generated the random allocation sequence electronically through a web-based randomisation system. The participants, sponsor, investigators, and nurses involved in the study were masked to the outcome of the randomisation and the randomisation key was only available to designated personnel at the hospital pharmacy. Masking was achieved through both identically appearing placebo tablets and concealment of the content of the tablets. Random assignment of patients was done in blocks of four, stratified according to the fibrosis stage at baseline and by self-reported alcohol abstinence within 6 months before inclusion. MI, BSM, and NT enrolled participants and assigned them to the trial groups.

### Procedures

We treated and followed up patients for 18 months from enrolment. The intervention consisted of oral tablet rifaximin-α 550 mg twice daily or a matching placebo, in line with current dosing recommendations of rifaximin-α as secondary prophylaxis for recurrent hepatic encephalopathy.^21^ Treatment duration was 18 months to enable detectable histological changes in liver fibrosis.^22^ We defined compliance to treatment as the ingestion of at least 75% of the planned treatment within the study period, assessed every 2 months by return of used blister packages. Self-reported alcohol intake was assessed at visits using predefined questions every 2 months. Patients were excluded from the study if they received antibiotics for 4 weeks or more during the study period, were non-compliant with the treatment, or withdrew their consent.

After 18 months of treatment, an end-of-study liver biopsy was done. Outcome assessment of the baseline and end-of-study biopsy samples was done by a single expert pathologist (SD) who was masked to treatment group and all clinical data. Quality requirements for liver biopsies were for them to be at least 10 mm long with at least six portal tracts or the presence of cirrhotic regenerative nodules. Liver fibrosis was assessed according to the Kleiner scoring system for fibrosis: F0, no fibrosis; F1, perisinusoidal or periportal fibrosis; F2, perisinusoidal and portal or periportal fibrosis; F3, bridging fibrosis; and F4, cirrhosis.^19^ Grading of lobular inflammation, ballooning, and steatosis was done according to the Non-Alcoholic Steatohepatitis (NASH) Clinical Research Network activity score.^19^

### Outcomes

The primary outcome was regression of liver fibrosis. This was defined as a between-treatment-group comparison of the proportion of patients who had a decrease of at least one fibrosis stage on the end of trial biopsy according to the Kleiner histological scoring system.^23^ Furthermore, we did a between-treatment-group comparison of the proportion of patients who had progression by at least one liver fibrosis stage.

Secondary histological endpoints were changes from baseline in lobular inflammation, hepatic steatosis, and hepatocyte ballooning. Non-invasive secondary endpoints were change in transient liver elastography, change in Fibrosis-4 (FIB-4) index, and change in N-terminal propeptide of type III collagen (PRO-C3), internal epitope in the 7S domain of type IV collagen (PRO-C4), and C-terminal of type VIII collagen (PRO-C8). The primary and secondary outcomes were all assessed at 18 months after enrolment. Gamma glutamyltransferase, alanine aminotransferase, and self-reported alcohol intake were assessed every second month after enrolment. Secondary outcomes of surrogate markers of fibrosis; hepatic stellate cell activation; blood and urine metabolomics; pro-inflammatory gene expression; quality of life; nutritional status; and gut microbiome composition and gene expression will be reported elsewhere (appendix p 2). Adverse events were classified according to the principles of the International Conference on Harmonization Good Clinical Practice guidelines.

### Statistical analysis

The sample size was based on a previous study on polyenylphosphatidylcholine in patients with alcohol-related liver disease in which 27% of participants regressed, 57% had stable disease, and 16% had progression by at least one fibrosis stage during a 2-year follow-up.^22^ However, because the study had a dropout rate of 48%, a true regression rate was assumed to be only half of the observed rate, corresponding to a true rate of regression of 14% during the trial period of 18 months. We considered a 25% difference in fibrosis regression as a clinically relevant difference. Accounting for an expected drop-out rate of 20%, α of 5%, and a power of 80%, 136 patients were needed in the study. There were no prespecified interim analyses for this study. Statistical programming commands for outcome analyses were performed masked and repeated after unmasking of treatment assignments as specified in the statistical analysis plan (appendix pp 45–60). According to the paired histopathological parameters, the outcomes were categorised as regression, stable, and progression, and dichotomised into regression versus no regression and progression versus no progression. Outcome analyses were done in both the per-protocol and modified intention-to-treat populations; safety was assessed in the full intention-to-treat population. The per-protocol population was defined as all randomly assigned patients who did not present serious protocol violations, who ingested at least 75% of the treatment and who were not withdrawn from the study due to non-adherence (interruption of treatment for 4 weeks or more). Patients receiving at least one dose of the intervention were included in the modified intention-to-treat analyses, and patients who did not complete the study (thereby having missing outcome data) were considered as having no response—in line with a previous study investigating the efficacy of semaglutide on hepatic inflammation and liver fibrosis assessed by liver histology in patients with non-alcoholic steatohepatitis.^24^ Deaths unrelated to liver disease or drug reaction were considered as missing. We did post-hoc sensitivity analyses excluding patients who could not have regression (F0), excluding patients who could not have progression (F4), and best-case and worst-case scenarios for the modified intention-to-treat population in which all patients who did not complete the study were considered as having regression and progression of liver fibrosis. Furthermore we did a post-hoc analysis comparing patients with at least two stage change in the fibrosis score. This was done in the whole study population and separately in the patients who could have such change (for regression, patients with F2–F4 at baseline; for progression, patients with F0–F2 at baseline). We also did a post-hoc subgroup analysis excluding patients with metabolic syndrome at baseline. Finally, we measured alcohol intake as assessed by phosphatidyl ethanol. We report baseline data as counts and frequencies and medians with IQRs. Comparison of binary outcome data are reported as odds ratio (OR) with 95% CI, and continuous outcome data are reported as estimated mean difference from baseline to 18 months with 95% CI. Unless noted otherwise, results are reported after adjustment for stratification factors (abstinence 6 months before inclusion and baseline fibrosis stage) using logistic and linear regression analysis accordingly. Sensitivity analyses were applied to adjust for age and sex. We considered two-sided p less than 0·05 as statistically significant and used STATA (version 17) for all analyses. This study is registered with The European Union Clinical Trials Register, EudraCT 2014–001856-51.

### Role of the funding source

The funders of the study had no role in study design, data collection, data analysis, data interpretation, or writing of the report.

## Results

**Figure 1:**
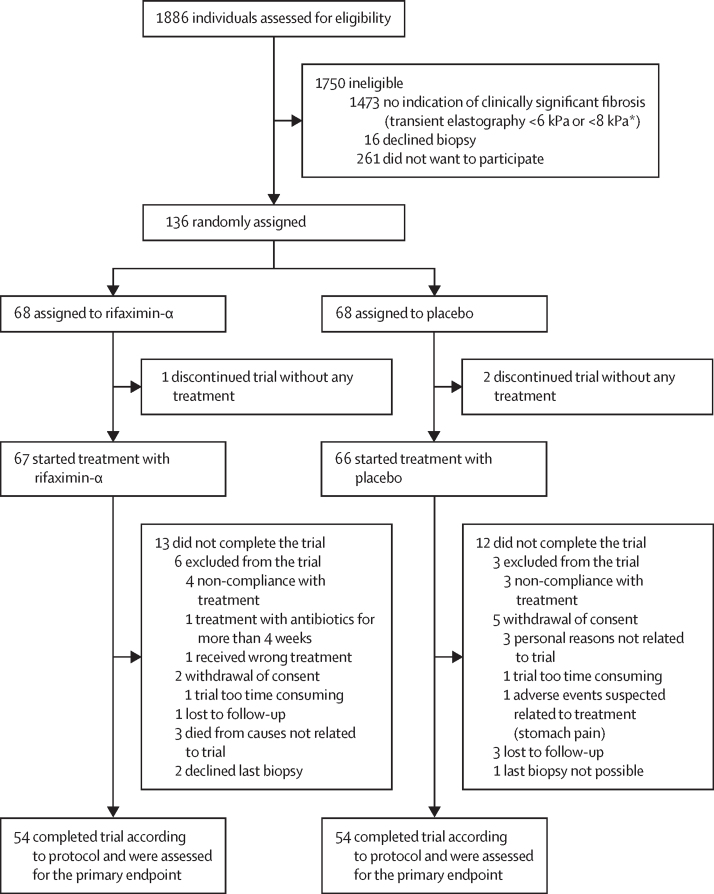
Trial profile *Threshold for liver biopsy according to transient elastography was increased during the study period due to increasing knowledge of cutoffs for ruling out clinically significant liver fibrosis. We excluded liver fibrosis in 398 patients using transient elastography cutoff of 6 kPa.^17^, ^30^ We excluded clinically significant liver fibrosis in 1075 patients using transient elastography cutoff of 8 kPa.

Between March 23, 2015, and Nov 10, 2021, we screened 1886 consecutive patients with a history of excessive alcohol consumption and identified 413 eligible for inclusion (figure 1). Of these patients, 136 consented to participate, were stratified according to fibrosis stage and alcohol abstinence within the previous 6 months, and were randomly assigned to rifaximin-α (n=68) or placebo (n=68). 133 (98%) patients received at least one dose of the intervention (67 patients in the rifaximin-α group and 66 patients in the placebo group), and comprised the modified intention-to-treat group. 108 (79%) patients completed the trial and were compliant to treatment per the protocol. All biopsy samples met the quality requirements.

At baseline, demographics and clinical characteristics between the rifaximin-α group and the placebo group were similar (table 1). All patients were White (100%), 114 (84%) were men, 22 (16%) were women, and the median age was 60 years (IQR 54–66). 16 (12%) patients reported being abstinent for at least 6 months before inclusion, 13 (81%) of whom reported abstinence throughout the trial. The median daily alcohol consumption for active drinkers was 51 g per day (IQR 34–86). Seven (5%) patients had no fibrosis (F0), 37 (27%) had stage F1, 63 (46%) had stage F2, 23 (17%) had stage F3, and six (4%) had stage F4 (table 1; appendix p 10); the median liver stiffness was 8·6 kPa (IQR 6·5–11·8). Characteristics of patients who did not complete the trial per protocol were similar between the groups and to those who did complete the trial (appendix p 3).

**Table 1:** Demographic and baseline clinical characteristics

			**Rifaximin-α group (n=68)**	**Placebo group (n=68)**
Age, years			60 (53–64)	60 (54–67)
Sex				
	Male		55 (81%)	59 (87%)
	Female		13 (19%)	9 (13%)
White race			68 (100%)	68 (100%)
Weight, kg			89·0 (73·4–95·6)	94·5 (83·0–108·1)
BMI, kg/m^2^			29·0 (25·5–31·6)	30·9 (27·0–33·9)
Smoking				
	Current		27 (40%)	26 (38%)
	Previous		18 (26%)	24 (35%)
	Never		23 (34%)	18 (26%)
Comorbidities				
	Type 2 diabetes		11 (16%)	14 (21%)
	Hypertension		29 (43%)	26 (38%)
	Hypertriglyceridaemia*		28 (41%)	33 (49%)
	Low HDL cholesterol†		15 (22%)	17 (25%)
Alcohol consumption				
	Alcohol abstinence within the previous 6 months, yes		8 (12%)	8 (12%)
	Daily alcohol consumption if not abstinent, g		51 (36–84)	51 (34–96)
	Phosphatidylethanol, μmol/L		0·69 (0·06–2·60)	0·76 (0·06–1·40)
	Years of excessive alcohol use			
		1–5 years	6 (9%)	10 (15%)
		6–10 years	13 (19%)	6 (9%)
		11–20 years	19 (28%)	19 (28%)
		21–30 years	16 (24%)	15 (22%)
		>30 years	14 (21%)	17 (25%)
Liver parameters				
	Alanine aminotransferase, U/L		38 (25–55)	39 (26–61)
	Aspartate aminotransferase, U/L		38 (24–64)	37 (26–53)
	Gamma glutamyl transferase, U/L		93 (41–237)	92 (52–289)
	Alkaline phosphatase, U/L		77 (67–99)	82 (70–99)
	Bilirubin, μmol/L		10 (7–14)	11 (7–14)
	Platelet count, 10^9^/L		224 (179–263)	222 (188–254)
	International normalised ratio		1·00 (0·97–1·09)	1·00 (0·90–1·07)
	Albumin, g/L		43 (41–45)	43 (41–47)
Liver histology				
	Fibrosis stage			
		0	3 (4%)	4 (6%)
		1	17 (25%)	20 (29%)
		2	33 (49%)	30 (44%)
		3	11 (16%)	12 (18%)
		4	4 (6%)	2 (3%)
	Lobular inflammation score			
		0	7 (10%)	6 (9%)
		1	40 (59%)	35 (51%)
		2	17 (25%)	25 (37%)
		3	4 (6%)	2 (3%)
	Ballooning score			
		0	42 (62%)	38 (56%)
		1	20 (29%)	25 (37%)
		2	6 (9%)	5 (7%)
	Steatosis grade			
		0	23 (34%)	17 (25%)
		1	19 (28%)	23 (34%)
		2	16 (24%)	21 (31%)
		3	10 (15%)	7 (10%)
Non-invasive test of liver fibrosis and steatosis				
	Liver stiffness measured by transient elastography, kPa		8·5 (6·5–11·8)	8·6 (6·3–11·6)
	Liver steatosis measured by controlled attenuation parameter, dB/m		312 (268–340)	318 (273–363)
	Fibrosis-4 score		1·6 (1·2–2·5)	1·7 (1·1–2·3)

Data are median (IQR) or n (%). The sum of percentages might deviate from 100 due to rounding.

* Hypertriglyceridaemia defined as triglycerides ≥1·7 mmol/L.

† Low HDL cholesterol defined as low if <1·03 mmol/L for men and <1·29 mmol/L for women.

**Figure 2:**
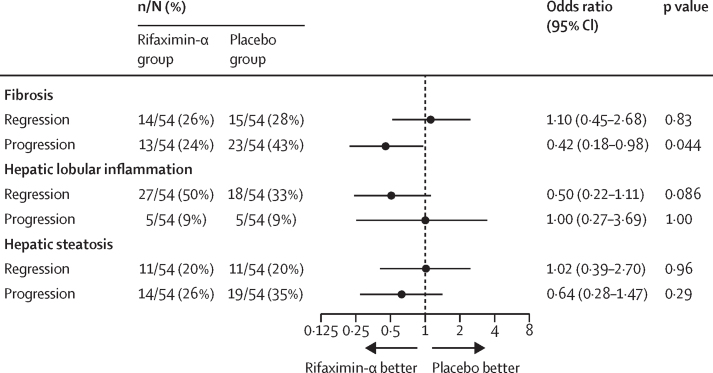
Forest plot of histological endpoints in the per-protocol population Liver biopsies were scored according to the scoring system designed by the Pathology Committee of the Non-Alcoholic Steatohepatitis Clinical Research Network.

In the per-protocol population, the proportion of patients with a decrease in liver fibrosis stage was similar in both groups (14 [26%] of 54 patients in the rifaximin-α group *vs* 15 [28%] of 58 patients in the placebo group; OR 1·10 [95% CI 0·45–2·68]; p=0·83; figure 2 and appendix p 11). The results were consistent in the modified intention-to-treat analyses, showing no effect of rifaximin-α on regression of liver fibrosis compared with placebo (15 [22%] of 67 patients in the rifaximin-α group and 15 [23%] of 66 patients in the placebo group; 1·05 [0·45–2·44]; p=0·91; appendix p 12).

In the per-protocol population, the proportion of patients with an increase in liver fibrosis stage was significantly lower in the rifaximin-α group compared to the placebo group (13 [24%] patients in the rifaximin-α group *vs* 23 [43%] patients in the placebo group; OR 0·42 [95% CI 0·18–0·98]; p=0·044; figure 2), corresponding to a number needed to treat of six (95% CI 3–92). The results were consistent in the modified intention-to-treat analyses, showing lower progression rates in the rifaximin-α group than the placebo group (13 [19%] patients in the rifaximin-α group *vs* 23 [35%] patients in the placebo group; adjusted OR 0·45 [0·20–1·02]; p=0·055; appendix p 12).

In the per-protocol population, there was no significant difference between the groups in the proportion of patients with a decrease in hepatic lobular inflammation; however, there was a larger number of patients in the rifaximin-α group than in the placebo group who had a reduction in hepatic lobular inflammation (figure 2 and appendix p 11). There was also no significant difference between the groups in the proportion of patients with increased hepatic lobular inflammation (figure 2). Similar results were observed in the modified intention-to-treat analysis (appendix p 12). Rifaximin-α had no significant effect on hepatic steatosis or ballooning compared with placebo in either the per-protocol or modified intention-to-treat analysis (figure 2 and appendix pp 4, 11).

**Figure 3:**
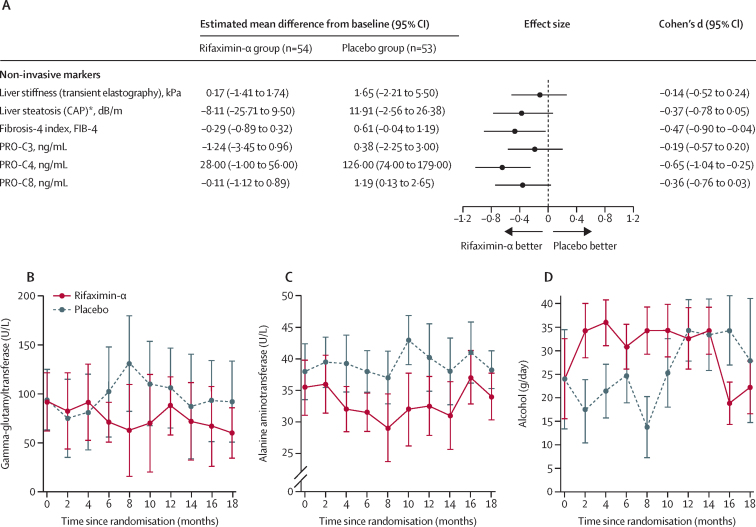
Changes in non-invasive fibrosis markers, liver enzymes, and self-reported alcohol consumption for the per-protocol population (A) Forest plot of effect sizes and corresponding 95 % CIs for changes in non-invasive secondary endpoints between baseline and 18 months. (B) Median gamma-glutamyltransferase concentrations over time. C) Median alanine aminotransferase concentrations over time. D) Median self-reported daily alcohol consumption over time. *Data from 91 patients (rifaximin-α n=46; placebo n=45). CAP=controlled attenuation parameter. PRO-C3=N-terminal propeptide of type III collagen. PRO-C4=internal epitope in the 7S domain of type IV collagen. PRO-C8=C-terminal of type VIII collagen.

Changes in non-invasive markers between baseline and 18 months of follow-up in the per-protocol population are shown in figure 3A and appendix p 8. Changes in FIB-4 index and PRO-C4 were significantly different between the rifaximin-α group and the placebo group at the 18-month follow-up; there was no significant difference between the groups in change in liver stiffness, liver steatosis, PRO-C3, or PRO-C8. In general, liver enzyme concentrations also changed in a more beneficial way in the rifaximin-α group than the placebo group (figure 3B–C). During the first 6 months of intervention, daily alcohol consumption was higher in the rifaximin-α group than the placebo group (figure 3D).

The most common reported adverse events were gastrointestinal disorders (including diarrhoea, abdominal pain, abdominal distension, and vomiting), infections, and trauma (table 2). In the rifaximin-α group, 48 (71%) of 68 patients reported an adverse or serious adverse event compared with 53 (78%) of 68 in the placebo group. All adverse events are reported in the appendix (p 9).

**Table 2:** Selected adverse events

		**Rifaximin-α group (n=68)**	**Placebo group (n=68)**
Any adverse events		48 (71%)	53 (78%)
Adverse events from gastrointestinal disorders system organ class		26 (38%)	32 (47%)
Adverse events from any system organ class, according to preferred term*			
	Diarrhoea	6 (9%)	12 (18%)
	Abdominal pain	6 (9%)	5 (7%)
	Abdominal distension	10 (15%)	6 (9%)
	Vomiting	1 (1%)	4 (6%)
	Infection	9 (13%)	8 (12%)
	Trauma	7 (10%)	8 (12%)
	Neck and back pain	1 (1%)	6 (9%)
Adverse events that resulted in premature discontinuation of treatment			
	All adverse events	0	1 (1%)
	Abdominal pain	0	1 (1%)
Serious adverse events			
	All serious adverse events	14 (21%)	12 (18%)
	Gastrointestinal disorders	4 (6%)	0
	Cardiovascular disorders	0	2 (3%)
	Respiratory, thoracic, and mediastinal disorders	0	1 (1%)
	Musculoskeletal and connective tissue disorders	2 (3%)	2 (3%)
	Infections	4 (6%)	0
	Renal and urinary disorders	0	1 (1%)
	Psychiatric disorders	0	1 (1%)
	Alcohol-related hospital admissions	2 (3%)	1 (1%)
	Liver-related event	2 (3%)	2 (3%)
	General disorders	0	2 (3%)
Neoplasms		1 (1%)	2 (3%)
	Malignant neoplasms	1 (1%)	1 (1%)
	Polyp in large intestine	0	1 (1%)
Fatal events		3 (4%)	0
	Small-cell lung carcinoma	1 (1%)	0
	Suicide	1 (1%)	0
	Sudden death (suspected cardiovascular disease)	1 (1%)	0

Data are number of patients with events (%).

* The most common adverse events with an incidence above 5% are reported here. Additional information on adverse events is in the appendix (p 9).

A serious adverse event occurred in 14 (21%) of 68 patients in the rifaximin-α group and 12 (18%) of 68 patients in the placebo group (table 2). The most common serious adverse events were gastrointestinal disorders and infections. No cases of infections with *Clostridioides difficile* or multidrug-resistant bacteria were reported. In the rifaximin-α group, no patients discontinued the treatment due to adverse events. In the placebo group, one patient discontinued the treatment after 3 months due to abdominal pain. No patients required dose reduction in the rifaximin-α group or placebo group. No serious adverse events were considered related to the study drug.

Three patients died during the trial (all in the rifaximin-α group), but none of the deaths were considered related to the study drug (table 2). One patient died of disseminated small-cell lung carcinoma, and one died due to suicide after a known psychiatric disorder. One sudden death, judged to be an acute myocardial infarction, occurred in a patient after 16 months of rifaximin-α with no previous adverse events.

In a per-protocol sensitivity analysis excluding patients who could not experience regression (F0 at baseline), the number of patients who had a decrease in liver fibrosis stage was 14 (27%) of 51 patients in the rifaximin-α group and 15 (30%) of 50 in the placebo group (OR 1·11 [95% CI 0·46–2·73]; p=0·80). In a per-protocol sensitivity analysis excluding patients who could not experience progression (F4 at baseline) the number of patients who had an increase in liver fibrosis stage was 13 (26%) of 50 patients in the rifaximin-α group and 23 (44%) of 52 in the placebo group (0·43 [0·18–1·01]; p=0·053). The results were robust when adjusted for age and sex (appendix pp 4–5). Results of the analyses of alternative best-case and worst-case scenarios in the modified intention-to-treat population are shown in the appendix (p 6). The number of patients with F2–F4 at baseline and a decrease of at least two stages was three (8%) of 38 in the rifaximin-α group and two (6%) of 34 in the placebo group (0·72 [0·11–4·65]; p=0·74). The number of patients with F0–F2 at baseline and an increase of at least two stages was one (2%) of 43 in the rifaximin-α group and seven (17%) of 41 in the placebo group (0·12 [0·01–0·99]; p=0·049). In the per-protocol population, a reduction of at least two stages of liver fibrosis was recorded in three (6%) of 54 patients in the rifaximin-α group and two (4%) of 54 patients in the placebo group (OR 0·61 [95% CI 0·10–4·08]; p=0·65), whereas progression of at least two stages of liver fibrosis occurred in one (2%) patient in the rifaximin-α group and seven (13%) patients in the placebo group (0·13 [0·01–1·07]; p=0·057; appendix pp 7, 13–14). A post-hoc subgroup analysis excluding patients with metabolic syndrome at baseline (13 in the rifaximin-α group and 21 in the placebo group) showed lower progression rate in the rifaximin-α group than the placebo group, with an effect size (OR 0·52 [95% CI 0·19–1·48]; p=0·22) similar to the entire study population. A post-hoc measurement of alcohol intake as assessed by phosphatidyl ethanol was similar between the groups and also with both progressors and non-progressors in the two treatment groups (appendix p 15).

## Discussion

GALA-RIF, an 18-month, randomised, double-blind, placebo-controlled trial, showed that rifaximin-α did not lead to regression of liver fibrosis in patients with biopsy-confirmed alcohol-related liver disease. However, rifaximin-α seemed to reduce progression of liver fibrosis, possibly due to attenuation of liver inflammation, despite a higher self-reported alcohol consumption in the rifaximin-α group during the trial. Our results suggest that rifaximin-α might be beneficial in patients with asymptomatic alcohol-related liver disease by preventing progression to decompensated cirrhosis.

Alcohol-related liver disease is the most prevalent cause of cirrhosis in the USA and Europe and liver fibrosis is the main prognostic factor contributing to liver-related morbidity and mortality.^1^, ^25^ Over the past decade, non-invasive tests such as transient elastography and blood-based biomarkers have allowed for early identification of liver fibrosis in patients with asymptomatic alcohol-related liver disease.^17^ This has facilitated a rapidly growing need for pharmaceutical treatments that can prevent progression to cirrhosis.^4^ In this trial, 550 mg rifaximin-α twice daily over 18 months significantly reduced the progression rate of liver fibrosis in the per-protocol population, with a number needed to treat of six. Furthermore, rifaximin-α seemed to reduce histological hepatic inflammation and improved non-invasive markers of fibrosis, inflammation, and extracellular matrix remodelling. The gut microbiome and gut-barrier function are altered in patients with alcohol-related liver disease and liver fibrosis.^26^ Results from this study support the hypothesis^8^ that the gut–liver axis is a modifiable target to stop progression of liver fibrosis in patients with asymptomatic alcohol-related liver disease.

In this study, progression of fibrosis over 18 months occurred in 43% of patients in the per-protocol placebo group, which is remarkably higher than the 16–21% over 2 years previously reported by Lieber and colleagues.^22^ The difference is probably related to the substantially lower drop-out rate in our study (21% *vs* 48%), which might provide a more accurate measure of the natural history of alcohol-related liver disease in a population with continuous drinking. Two observational cohort studies of patients with asymptomatic alcohol-related liver fibrosis reported incidences of hepatic decompensation of 18% over 49 months and 34% over 90 months.^3^, ^5^ This figure is more than three times higher than in patients with non-alcoholic steatohepatitis with liver fibrosis, most of whom also die from causes unrelated to liver disease.^27^ Evaluating the efficacy of interventions for patients with liver fibrosis should reflect this difference in the natural course of disease, and interventions that either halt progression or promote regression should both be considered of the highest importance.

Bacterial antimicrobial resistance is a major global burden, and long-term use of antibiotics, such as rifaximin-α, could be associated with the development of infections with multidrug-resistant bacteria and *C difficile.* In our study, no cases of multidrug-resistant bacteria and *C difficile* occurred and, in general, rifaximin-α was well tolerated with no increased risk of adverse events compared with placebo, which is consistent with the published literature.

This study shows that rifaximin-α is well tolerated and might reduce fibrosis progression. However, the starting point for the treatment of patients with alcohol-related liver disease should be alcohol rehabilitation. Unfortunately, most patients with an alcohol dependence do not achieve long-term abstinence, but have periods of relapse.^7^ In these patients, rifaximin-α could be used in drinking periods as a secondary prevention to avoid progression to higher stages of fibrosis and development of hepatic decompensation.^15^ A higher self-reported alcohol consumption was seen in the rifaximin-α group during the trial, supporting that the lower fibrosis progression rate in the rifaximin-α group is a true effect of the intervention and not caused by reduced drinking. We are not aware of any studies showing that rifaximin-α increases alcohol intake. During the trial period, three deaths were observed, of which none were suspected to be related to the intervention. No increased mortality risk has previously been described with rifaximin-α in patients with liver disease.^13^, ^14^, ^28^

Strengths of this study include the long intervention period and the histological assessment of liver fibrosis as the primary outcome supported by non-invasive markers. The high compliance in an otherwise vulnerable population strengthens the findings, but individuals with alcohol-related liver disease outside a clinical trial might not have the same compliance, which should be considered if implemented. The main limitation is that no significant difference was noted between groups for the primary endpoint, decrease of liver fibrosis. Preferably, the beneficial effect of rifaximin-α on halting fibrosis progression should be validated in a multicentre, phase 3, confirmatory efficacy trial with hard clinical endpoints. For the inclusion criteria, we used the histological assessment by the on-call pathologist at the hospital. During the masked outcome assessment by the single expert pathologist, seven patients were downgraded from F1 to F0 at baseline and could consequently not reach the primary outcome. Since the proportion of F0 patients was only 5%, and distribution of these patients was balanced between the groups (four *vs* three), it only had a minor effect on the estimated effects, which was supported by the sensitivity analysis. We included patients with a wide range of liver fibrosis severity (from no fibrosis to cirrhosis) and the therapeutic goal should perhaps be different depending on the severity of liver fibrosis and the risk of progression. Patients in this trial generally had substantial alcohol overuse, but no extreme alcohol overuse, and exhibited several metabolic risk factors. They therefore represent the majority of people who drink in excess.^2^, ^29^ Finally, all patients in this study were White, and rifaximin-α might work differently in other racial and ethnic groups. Future studies should address these issues.

In patients with biopsy-confirmed alcohol-related liver disease, 18 months of treatment with rifaximin-α did not promote regression of liver fibrosis, but it did appear to reduce fibrosis progression. These results suggest that rifaximin-α seems to decrease progression of liver fibrosis in patients with alcohol-related liver disease and therefore might be beneficial for individuals who cannot achieve alcohol abstinence.

## Data sharing

Data are available on request after approval from the Danish Data Protection Agency in a pseudonymised manner upon request to mads.israelsen@rsyd.dk. The study protocol and statistical analysis plan are available in the appendix (pp 18, 45).

## Declaration of interests

JKH has received speaking fees from Norgine. KPL has received speaking fees and support for travels from Siemens. MKj has received speaking fees from Siemens. MKa owns stock in Nordic Bioscience. MA has received speaking or consulting fees from Roche, Lundbeck Pharma, and SNIPR Biome. JT has received speaking or consulting fees from Versantis, Gore, Boehringer-Ingelheim, Alexion, Falk, Grifols, Genfit, and CSL Behring and participated in advisory boards for Versantis, Alexion, Sobi, Gore, Grifols, Genfit, and CSL Behring. MT has received speaking fees from Siemens Healthcare, Norgine, Echosens, and Tillotts Pharma; consulting fees from GE Healthcare; and participated in advisory boards for ID Liver and Alcohol & Society. AK has served as speaker for Norgine, Siemens, and Nordic Bioscience and participated in advisory boards for Norgine and Siemens, all outside the submitted work. AK receives royalties from Gyldendal and has received equipment, drugs, or other services from Norgine, Siemens, and Echosense. All other authors declare no competing interests.
