# Supplementary material for: Rifaximin-α for liver fibrosis in patients with alcohol-related liver disease (GALA-RIF): a randomised, double-blind, placebo-controlled, phase 2 trial
Source: Lancet Gastroenterol Hepatol. 2023 Mar 6;8(6):523–32. doi: 10.1016/S2468-1253(23)00010-9 (PMC10172147; doi:10.1016/S2468-1253(23)00010-9)
Supplement: Supplementary appendix [file mmc1.pdf]

THE LANCET
Gastroenterology & Hepatology

Supplementary appendix

This appendix formed part of the original submission and has been peer reviewed. We post it as supplied by the authors.

Supplement to: Israelsen M, Madsen BS, Torp N, et al. Rifaximin-α for liver fibrosis in patients with alcohol-related liver disease (GALA-RIF): a randomised, double-blind, placebo-controlled, phase 2 trial. *Lancet Gastroenterol Hepatol* 2023; published online March 6. https://doi.org/10.1016/S2468-1253(23)00010-9.

Appendix

PAGE 2	Table S1: Complete list of outcomes
PAGE 3	Table S2: Patient characteristics for patients who dropped out of the study
PAGE 4	Table S3: Effect of rifaximin-α on hepatocellular ballooning
PAGE 4	Table S4a: Sensitivity analysis on reduction of fibrosis stage
PAGE 5	Table S4b: Sensitivity analysis on progression of fibrosis stage
PAGE 6	Table S5: Best-case and worst-case mITT analyses
PAGE 7	Table S6: Fibrosis stages on the individual participant level
PAGE 8	Table S7: Differences in non-invasive markers from baseline to end of study
PAGE 9	Table S8: Adverse events by system organ class
PAGE 10	Figure S1: Distribution of Kleiner fibrosis stage at baseline
PAGE 11	Figure S2: Percentage of patients with histological endpoint changes
PAGE 12	Figure S3: Odds ratios for histological endpoints in modified intention-to treat analysis
PAGE 13	Figure S4: Changes in Kleiner fibrosis stage from baseline to end of study
PAGE 14	Figure S5. Waterfall plot. Individual changes in Kleiner fibrosis stage
PAGE 15	Figure S6. Alcohol intake assessed by phosphoidylethanol (PEth)
PAGE 16	CONSORT Checklist
PAGE 18	Protocol
PAGE 45	Statistical Analysis Plan

Appendix Tables

Table S1: Complete list of outcomes according to protocol

**Reported in current manuscript**	**Plan to be reported later**
**Primary**<br><br>Histology<br>- Liver fibrosis<br><br>**Secondary**<br><br>Histology<br>- Lobular inflammation<br>- Hepatic steatosis<br>- Hepatocyte ballooning<br><br>Non-invasive<br>- Transient liver elastography (TE)<br>- Fibrosis-4 index (FIB-4)<br>- N-terminal pro-peptide of type III collagen (PRO-C3)<br>- Internal epitope in the 7S domain of type IV collagen (PRO-C4)<br>- C-terminal of type VIII collagen (PRO-C8)<br>- Standard liver blood tests: Alanine aminotransferase (ALT) and gamma-glutamyl transferase (GGT)<br><br>Alcohol consumption<br>- Self-reported alcohol intake	**Secondary**<br><br>- Surrogate markers of fibrosis: collagen proportionate area, shear wave elastography, enhanced liver fibrosis score, hydroxylprolin level, TIMP-1, MMP2, key pro-fibrotic cytokines (TGF-β1, PDGF-β-R, CTGF) and key pro-inflammatory cytokines (TNF-α, MCP-1) and CD 163<br>- Shear wave elastography<br>- Activation of hepatic stellate cells estimated by a reduction in the area of α-smooth muscle actin positive cells<br>- Composition of the microbiota estimated by 454 pyrusequencing technology of faecal samples<br>- Gene expression of the microbiota estimated by transcriptomic of faecal samples<br>- Changes in the composition of the gut microbiota assessed by shotgun metagenomics sequencing<br>- Quality of life assessed by the Short Form (36) Health Survey and Chronic Liver Disease Questionnaire<br>- Nutritional status assessed by weight and hand grip strength<br>- Host pro-inflammatory gene expression in hepatic tissue estimated by transcriptomics and microRNA profiling<br>- Host metabolomics of blood and urine including micro-RNA profile

Table S2: Patient characteristics for patients who dropped out of the study.

Characteristics	Rifaximin-α (N=14)	Placebo (N=14)	p
Age, yrs.	58 (51-61)	61 (54-69)	0.12
Sex, n (male)	13 (93%)	10 (71%)	0.33
BMI, kg/m^2	27 (25-29)	29 (25-31)	0.62
Smoking, (current/previous/never)	7/4/3 (50%/29%/21%)	8/4/2 (57%/29%/14%)	0.65
**Comorbidities**			
Type 2 diabetes, n	2 (14%)	2 (14%)	1.00
Hypertension, n	5 (36%)	5 (36%)	1.00
**Alcohol consumption**			
Alcohol abstinence within the previous six months, (yes)	1 (7%)	0 (0%)	1.00
Daily alcohol consumption if not abstinent, g	76 (45-158)	42 (24-60)	0.13
Years of excessive alcohol use:<br>1-5 years<br>6-10 years<br>11-20 years<br>21-30 years<br>>30 years	 1 (7%)<br>1 (7%)<br>7 (50%)<br>5 (36%)<br>0 (0 %)	 3 (21%)<br>1 (7%)<br>4 (29%)<br>1 (7%)<br>5 (36%)	0.26
Liver histology			
Fibrosis (stage 0/1/2/3/4)	1/3/6/4/0	1/3/9/1/0	0.45
Lobular inflammation (score 0/1/2/3)	2/6/5/1	0/7/6/1	0.50
Ballooning (score 0/1/2)	8/4/2	8/5/1	0.88
Steatosis (grade 0/1/2/3)	1/5/4/4	1/4/6/3	0.98
Non-invasive test of liver fibrosis and steatosis			
Liver stiffness, measured by transient elastography (kPa)	8.1 (6.8-16.4)	8.5 (5.7-9.7)	0.79
Fibrosis-4 score	2.1 (1.2-5.5)	1.6 (1.3-4.7)	0.31
All summary data are medians (25%-75% percentile) or counts (%)<br>The sum of percentages may deviate from 100 due to rounding			

Table S3: Effect of rifaxmin-α on hepatocellular ballooning assessed by liver histology. Results are obtained from a multivariable logistic analysis.

	Rifaxmin-α n/N (%)	Placebo n/N (%)	Odds ratio	95% CI	P-value
PP-analysis					
Regression	10/54 (19%)	9/54 (17%)	0.89	0.33 to 2.40	0.81
Progression	6/54 (11%)	7/54 (13%)	0.84	0.26 to 2.69	0.767
mITT-analysis					
Regression	10/67 (15%)	9/66 (14%)	0.91	0.34 to 2.43	0.86
Progression	6/67 (9%)	7/66 (11%)	0.83	0.26 to 2.61	0.75
Results are obtained from multivariable logistic analysis after adjustment for stratification factors (abstinence 6 months before inclusion and baseline fibrosis stage).					

Table S4a: Sensitivity analysis on the primary outcome reduction of fibrosis stage. Results are obtained from a multivariable logistic analysis.

	Odds ratio	95% CI	P-value
PP-analysis			
Rifaxmin-α (yes)	1.03	0.41 to 2.62	0.947
Fibrosis stage, baseline	2.12	1.11 to 4.07	0.024
Abstinence at inclusion (yes)	2.74	0.83 to 9.06	0.098
Sex (male)	0.81	0.21 to 3.10	0.759
Age (year)	0.94	0.89 to 0.99	0.022
mITT-analysis			
Rifaxmin-α (yes)	1.05	0.44 to 2.51	0.913
Fibrosis stage, baseline	1.94	1.04 to 3.63	0.037
Abstinence (yes)	3.35	1.06 to 10.57	0.040
Sex (male)	0.86	0.24 to 3.13	0.823
Age (year)	0.94	0.90 to 0.99	0.016

Table S4b: Sensitivity analysis on the progression of fibrosis stage. Results are obtained from multivariable logistic analysis.

	Odds ratio	95% CI	P-value
PP-analysis			
Rifaxmin-α (yes)	0.38	0.16 to 0.93	0.033
Fibrosis stage, baseline	1.90	1.04 to 3.48	0.036
Abstinence at inclusion (yes)	1.39	0.38 to 5.03	0.620
Sex (male)	1.11	0.35 to 3.55	0.862
Age (year)	0.96	0.91 to 1.01	0.135
mITT-analysis			
Rifaxmin-α (yes)	0.46	0.20 to 1.03	0.059
Fibrosis stage, baseline	2.00	1.11 to 3.59	0.021
Abstinence (yes)	1.07	0.30 to 3.72	0.921
Sex (male)	0.80	0.34 to 2.38	0.689
Age (year)	0.97	0.93 to 1.02	0.273

Table S5: Results from the best-case mITT analysis and the worst-case mITT analysis.

	Odds ratio	95% CI	P-value
**Best-case**			
Improvement			
Rifaxmin-α (yes)	0.96	0.48 to 1.93	0.91
Worsening			
Rifaxmin-α (yes)	0.45	0.20 to 1.02	0.055
**Worst-case**			
Improvement			
Rifaxmin-α (yes)	1.05	0.45 to 2.44	0.91
Worsening			
Rifaxmin-α (yes)	0.56	0.28 to 1.12	0.10
In the best-case mIIT, it was assumed that all patients having missing end-of-study biopsy have improvement of liver fibrosis according to a regression rate of 27 (40%) in the rifaximin-α group vs. 26 (39%) in the placebo group. In the worst-case mIIT, it was assumed that all patients having missing end-of-study biopsy have progression of liver fibrosis according to a progression rate in the rifaximin-α group of 25 (37%) vs. 34 (52%) in the placebo group.			

Table S6: Overview of the fibrosis stages at baseline and after 18 months of intervention at the individual participant level.

Rifaxmin-α

		18 months						
		**F0**	**F1**	**F2**	**F3**	**F4**	**Missing**	**Total**
Baseline	**F0**	2	0	0	0	0	1	3
	**F1**	2	7	5	0	0	3	17
	**F2**	3	8	10	6	1	5	33
	**F3**	0	1	1	4	1	4	11
	**F4**	0	0	0	0	4	0	4
	**Total**	7	16	16	10	6	13	68

Placebo

		18 months						
		**F0**	**F1**	**F2**	**F3**	**F4**	**Missing**	**Total**
baseline	**F0**	0	2	1	0	0	1	4
	**F1**	2	8	6	2	0	2	20
	**F2**	1	5	6	5	4	9	30
	**F3**	0	1	4	3	3	1	12
	**F4**	0	0	0	2	0	0	2
	**Total**	3	16	17	12	7	13	68

Table S7: Changes from baseline to end of study in non-invasive markers according to treatment group.

	Estimated Mean Difference From Baseline (95% CI)			
	Rifaximin-α (n=54)	Placebo (n=53)	Mean Difference in Change	P-value
Liver stiffness (TE), kPa	+0.17<br>(-1.41 to +1.74)	+1.65<br>(-2.21 to +5.50)	-1.48<br>(-5.51 to 2.10)	0.46
Liver Steatosis (CAP)*, dB/m	-8<br>(-26 to +9)	+12<br>(-3 to +26)	-20<br>(-42 to +3)	0.081
Fibrosis-4 index (FIB-4)	-0.29<br>(-0.89 to +0.32)	+0.61<br>(-0.04 to +1.19)	-0.90<br>(-1.72. to -0.07)	0.033
PRO-C3 (ng/mL)	-1.24<br>(-3.45 to +0.96)	+0.38<br>(-2.25 to +3.00)	-1.62<br>(-5.03 to +1.79)	0.35
PRO-C4 (ng/mL)	+28<br>(-1 to +56)	+126<br>(+74 to +179)	-98<br>(-158 to -39)	0.0015
PRO-C8 (ng/mL)	-0.11<br>(-1.12 to +0.89)	+1.19<br>(0.13 to +2.65)	-1.50<br>(-3.11 to 0.10)	0.066

*Based on data from 91 patients
PRO-C3, N-terminal pro-peptide of type III collagen; PRO-C4, Internal epitope in the 7S domain of type IV collagen; PRO-C8, C-terminal of type VIII collagen.

Table S8: Adverse events by system organ class.

	Rifaximin-α (N=68)	Placebo (N=68)
**Any adverse or serious adverse events**	48 (71%)	53 (78%)
Gastrointestinal disorders	26 (38%)	32 (47%)
General disorders and administration site conditions	9 (13%)	15 (22%)
Musculoskeletal and connective tissue disorders	3 (4%)	12 (18%)
Infections and infestations	9 (13%)	10 (15%)
Injury, poisoning, and procedural complications	10 (15%)	8 (12%)
Skin and subcutaneous tissue disorders	2 (3%)	6 (9%)
Respiratory, thoracic, and mediastinal disorders	1 (1%)	4 (6%)
Psychiatric disorders	4 (6%)	0
Endocrine disorders	2 (3%)	3 (4%)
Alcohol-related disorders	3 (4%)	2 (3%)
Metabolism and nutrition disorders	2 (3%)	1 (1%)
Renal and urinary disorders	2 (3%)	1 (1%)
Cardiovascular disorders	2 (3%)	1 (1%)
Reproductive system and breast disorders	0	2 (3%)
Immune system disorders	1 (1%)	1 (1%)
Neoplasms benign, malignant, and unspecified	1 (1%)	0
Nervous system disorders	0	1 (1%)
Blood and lymphatic system disorders	0	1 (1%)
Eye disorders	0	1 (1%)

Data are shown as number of events with incidences in parenthesis.
Serious adverse events and adverse events that resulted in premature discontinuation of the treatment are shown in Table 2.

Supplementary Figures

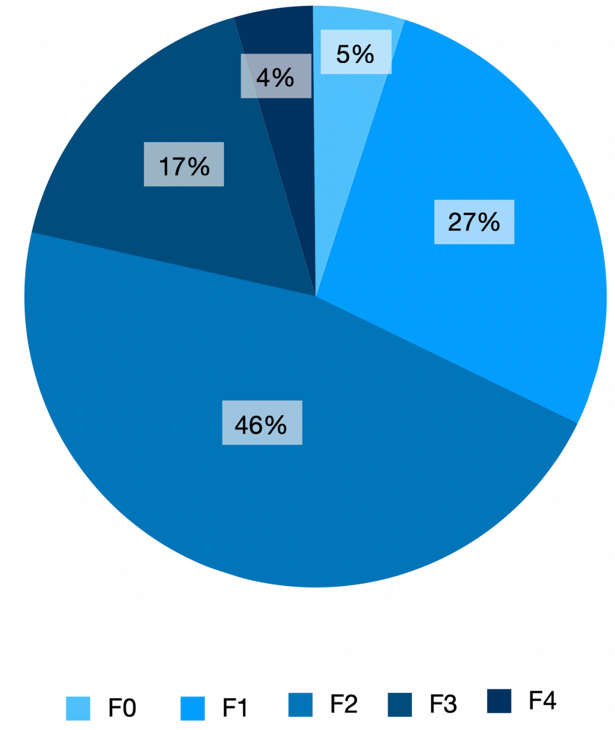

Figure S1. Distribution of Kleiner fibrosis stage at baseline.
The percentage of patients at baseline with Kleiner fibrosis stage F0 (no fibrosis), F1 (perisinusoidal or periportal fibrosis), F2 (perisinusoidal and portal/periportal fibrosis), F3 (bridging fibrosis), or F4 (cirrhosis).

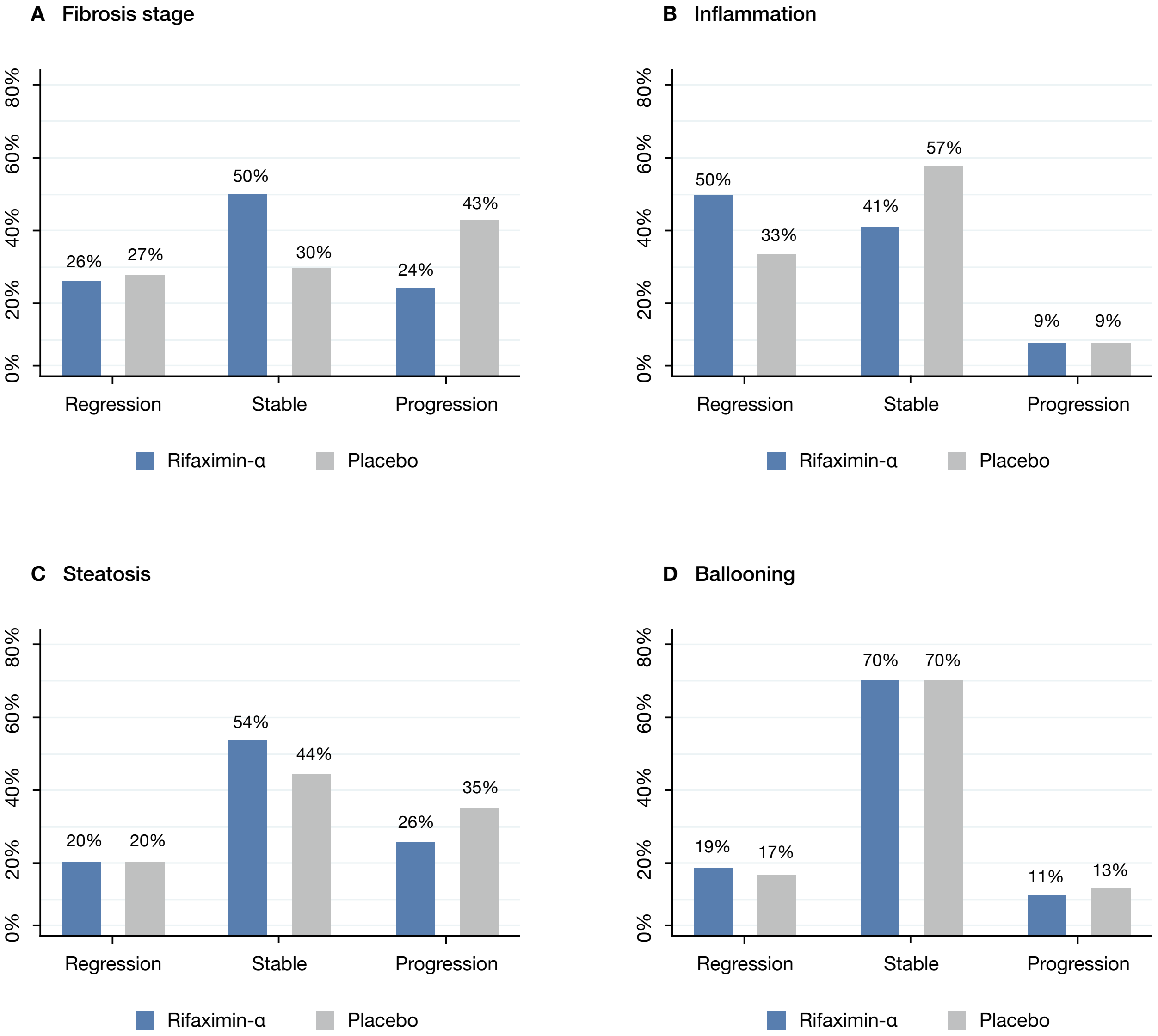

Figure S2. Percentage of patients with histological endpoint changes
The percentage of patients who experienced regression, were stable, or had progression according to A) fibrosis stage, B) inflammation, C) steatosis, and D) ballooning.

Endpoint	Rifaximin group	Control group	Odds Ratio (95% CI)
	no. of events/total no. (%)		
**Fibrosis**			
Regression vs no regression	15/67 (22)	15/66 (23)	1.05 (0.45-2.44)
Progression vs no progression	13/67 (19)	23/66 (35)	0.45 (0.20-1.02)
**Inflammation**			
Regression vs no regression	27/67 (40)	19/66 (29)	0.61 (0.29-1.26)
Progression vs no progression	5/67 (7)	5/66 (8)	1.00 (0.27-3.63)
**Steatosis**			
Regression vs no regression	11/67 (16)	12/66 (18)	1.18 (0.47-2.94)
Progression vs no progression	15/67 (22)	19/66 (29)	0.71 (0.32-1.56)

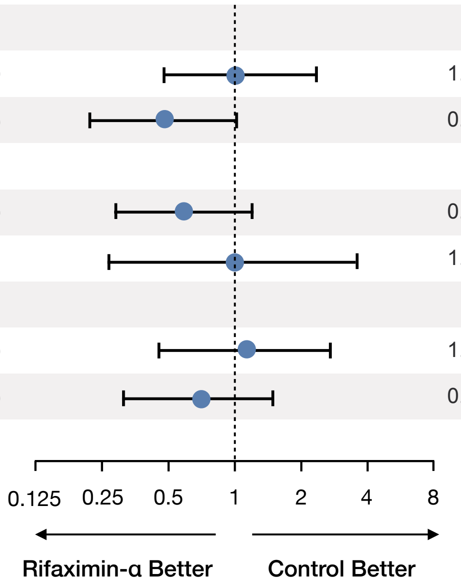

Figure S3. Odds ratios for histological endpoints — modified intention-to-treat analysis.
Odds ratios with 95% confidence intervals for the histological endpoints; fibrosis, inflammation and steatosis. Liver biopsies were scored according to the scoring system designed by the Pathology Committee of the Non-Alcoholic Steatohepatitis Clinical Research Network (NASH CRN). Data is from a modified intention-to-treat analysis. The dashed line indicates an odds ratio of 1.0.

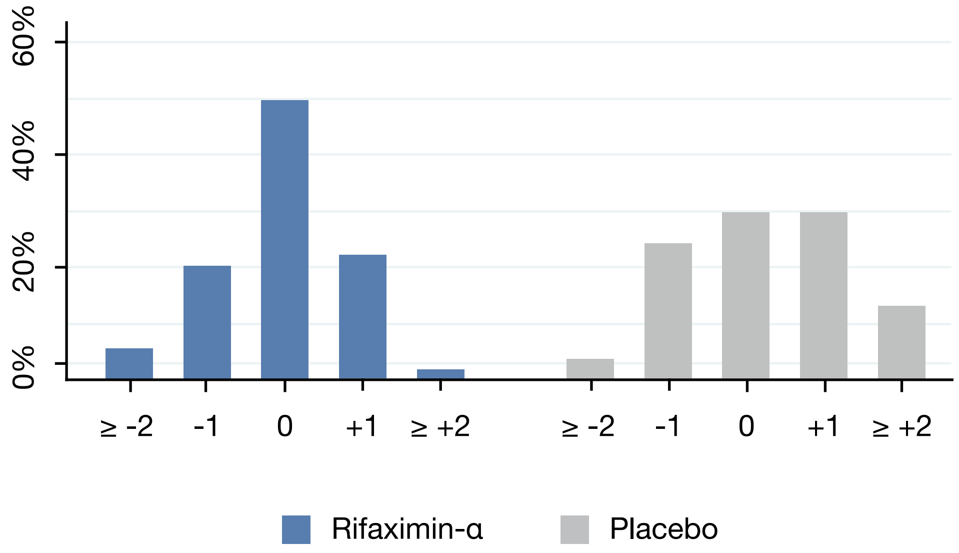

Figure S4. Changes in Kleiner fibrosis stage.
The percentage of patients with changes in Kleiner fibrosis stage from baseline to end of study.

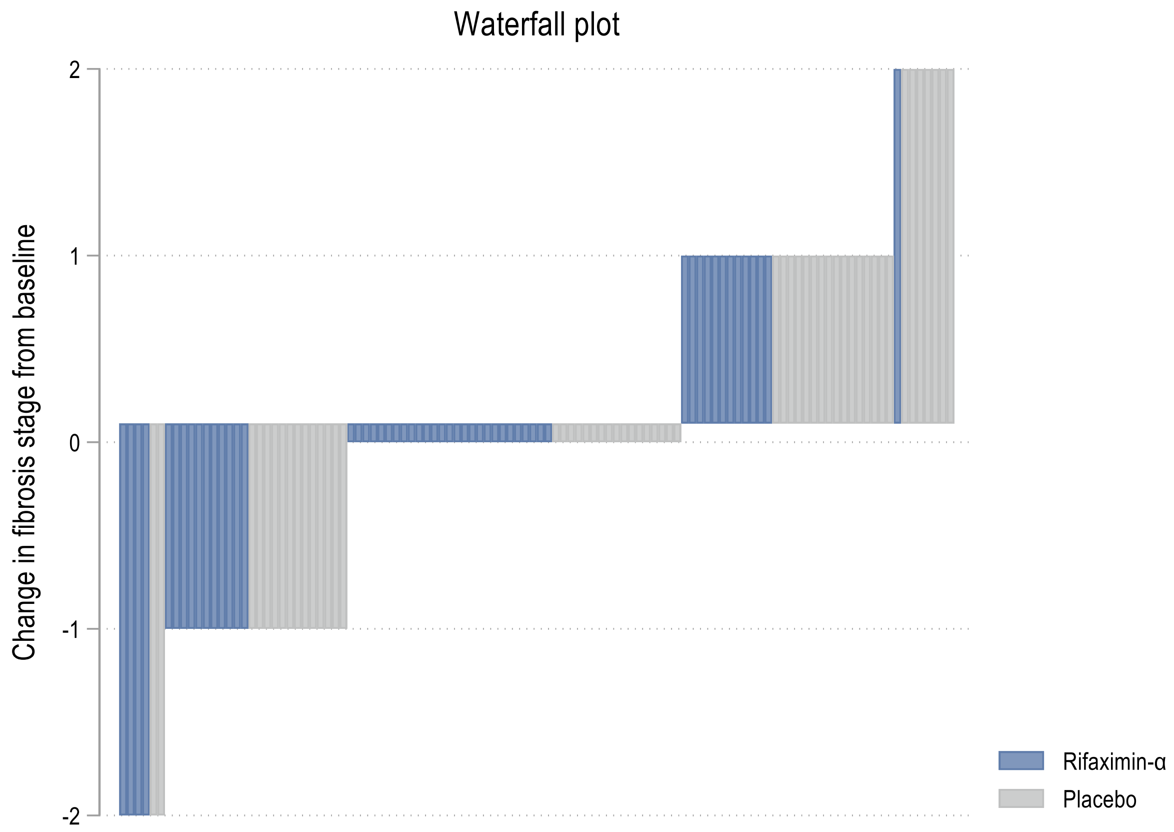

Figure S5. Waterfall plot. Individual changes in Kleiner fibrosis stage.
Number of patients from per protocol population (n=108) with changes in Kleiner fibrosis stage from baseline until end of study.

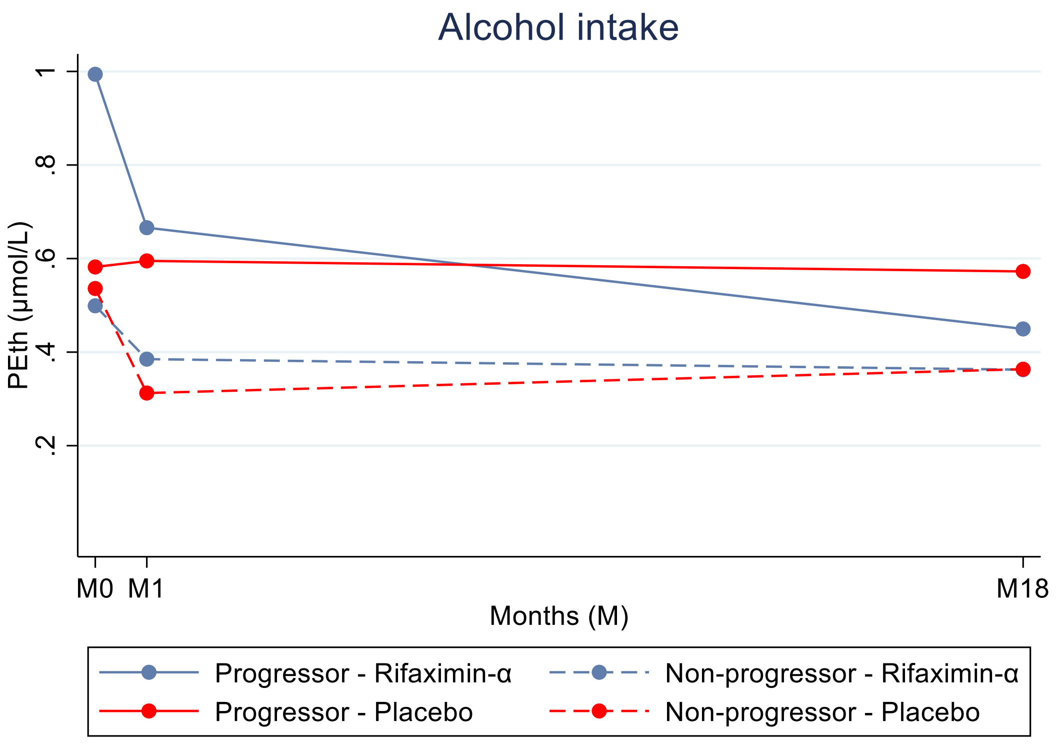

Figure S6. Alcohol intake assessed by phosphoidylethanol (PEth)
Changes in alcohol consumption as measured by PEth at start of treatment (M0), after one month (M1) and at end of treatment at 18 months (M18).

CONSORT 2010 checklist

Section/Topic	Item no.	Checklist item	Reported on page no.
**Title and abstract**			
	1a	Identification as a randomizsed trial in the title	1
	1b	Structured summary of trial design, methods, results, and conclusions (for specific guidance see CONSORT for abstracts)	3-5
**Introduction**			
Background and objectives	2a	Scientific background and explanation of rationale	8
	2b	Specific objectives or hypotheses	8-9
**Methods**			
Trial design	3a	Description of trial design (such as parallel, factorial) including allocation ratio	10
	3b	Important changes to methods after trial commencement (such as eligibility criteria), with reasons	11
Participants	4a	Eligibility criteria for participants	10-11
	4b	Settings and locations where the data were collected	10
Interventions	5	The interventions for each group with sufficient details to allow replication, including how and when they were actually administered	12
Outcomes	6a	Completely defined pre-specified primary and secondary outcome measures, including how and when they were assessed	12-13 + Appendix p. 2
	6b	Any changes to trial outcomes after the trial commenced, with reasons	11
Sample size	7a	How sample size was determined	13
	7b	When applicable, explanation of any interim analyses and stopping guidelines	NA
Randomisation:			
Sequence generation	8a	Method used to generate the random allocation sequence	11
	8b	Type of randomisation; details of any restriction (such as blocking and block size)	11
Allocation concealment mechanism	9	Mechanism used to implement the random allocation sequence (such as sequentially numbered containers), describing any steps taken to conceal the sequence until interventions were assigned	11
Implementation	10	Who generated the random allocation sequence, who enrolled participants, and who assigned participants to interventions	11-12
Blinding	11a	If done, who was blinded after assignment to interventions (for example, participants, care providers, those assessing outcomes) and how	11--13
	11b	If relevant, description of the similarity of interventions	NA
Statistical methods	12a	Statistical methods used to compare groups for primary and secondary outcomes	13-14
	12b	Methods for additional analyses, such as subgroup analyses and adjusted analyses	14
**Results**			
Participant flow (a diagram is strongly recommended)	13a	For each group, the numbers of participants who were randomly assigned, received intended treatment, and were analysed for the primary outcome	15 + Fig. 1
	13b	For each group, losses and exclusions after randomisation, together with reasons	15 + Fig. 1
Recruitment	14a	Dates defining the periods of recruitment and follow-up	15
	14b	Why the trial ended or was stopped	15
Baseline data	15	A table showing baseline demographic and clinical characteristics for each group	Table 1
Numbers analysed	16	For each group, number of participants (denominator) included in each analysis and whether the analysis was by original assigned groups	15-17
Outcomes and estimation	17a	For each primary and secondary outcome, results for each group, and the estimated effect size and its precision (such as 95% confidence interval)	15-17
	17b	For binary outcomes, presentation of both absolute and relative effect sizes is recommended	15-17

Ancillary analyses	18	Results of any other analyses performed, including subgroup analyses and adjusted analyses, distinguishing pre-specified from exploratory	16-17
Harms	19	All important harms or unintended effects in each group (for specific guidance see CONSORT for harms)	18
**Discussion**			
Limitations	20	Trial limitations, addressing sources of potential bias, imprecision, and, if relevant, multiplicity of analyses	21
Generalisability	21	Generalisability (external validity, applicability) of the trial findings	19-21
Interpretation	22	Interpretation consistent with results, balancing benefits and harms, and considering other relevant evidence	19-21
**Other information**			
Registration	23	Registration number and name of trial registry	3
Protocol	24	Where the full trial protocol can be accessed, if available	Appendix
Funding	25	Sources of funding and other support (such as supply of drugs), role of funders	14

Anti-fibrotic and molecular aspects of rifaximin in alcoholic liver disease: A randomized placebo controlled clinical trial

September, 2019

Eudra CT number: 2014-001856-51

Sponsor:

Professor, PhD, Aleksander Krag
Department of Medical Gastroenterology
Odense University Hospital
Sdr. Boulevard 29
5000 Odense C
Tlf: +4521409915
Mail: aleksander.krag@rsyd.dk

Principle investigator:

MD, PhD, Mads Bastrup Israelsen
Department of Medical gastroenterology
Odense University Hospital
Sdr. Boulevard 29
5000 Odense C
Tlf:+45 51 82 99 58
Mail: mads.egerod.israelsen@rsyd.dk

Table of content

1 Primary centre of investigations ... 21
2 Collaborating unit ... 21
3 Sub investigators: ... 22
4 Supervisors of the PhD Thesis ... 22
5 Introduction ... 24
5.1 Microbiota induced liver fibrosis ... 24
5.2 Targeting the intestinal microbiota ... 24
6.0 Aim and hypothesis of the study ... 25
6.1 Primary outcome: ... 25
6.2 Secondary outcomes: ... 25
7.0 Study design ... 25
7.1 Inclusion criteria ... 26
7.2 Exclusion criteria ... 26
8.0 Withdrawal from the study ... 27
9.0 Dropout ... 27
10 Recruitment and enrolment of participants ... 27
11 Time schedule: ... 27
12 Drug information ... 27
13 Side effects, risks and disadvantages related to medication ... 28
14 Possible benefits of rifaximin for trial participants ... 30
15 Handling of trial medication ... 30
16 Record keeping of trial medication ... 30
17 Randomisation methods ... 31
18 Disruption of blinding ... 31
19 Adverse events and side effects ... 32
20 Registration of adverse event/adverse reaction ... 32
20.1 Reporting of Adverse Events (AE) ... 33
20.2 Reporting of Serious Adverse Events (SAE) ... 33
20.3 Reporting of Suspected Unexpected Serious Adverse Reaction (SUSAR) ... 33
21 Measurements and investigations ... 33
Investigations day 0 ... 33

Investigations 1 month 34
Investigations 2,4,6,8,10,12,14,16 Month 34
Investigations 18 Month 34
Optional Follow up meeting 19 Month 35
22 Risks and disadvantages related to investigations and measurements 35
23 Establishment of a Bio bank 35
24 Data management 36
25 Statistics 36
26.0 Ethics 37
26.1 Obtaining consent 37
26.2 Patient disadvantages and risk 38
26.3 Patient Benefit 38
27 Insuring and Financing 38
28 Publication 40
29 Implications 40
Appendix 1:Standard operational procedures for shear wave elastography with *Supersonic Aixplorer.* .. 43
Appendix 2:Standard operational procedures for Transient elastography of liver and spleen using FibroScan (Echosens, France) 44

1 Primary centre of investigations

Department of Medical gastroenterology
Odense University Hospital
Sdr. Boulevard 29
5000 Odense C
Tel:65412755

2 Collaborating unit

Analysis and investigations:

Biochemistry:
Afdelingen for klinisk biokemi og farmakologi
Sdr. Boulevard 29
5000 Odense C
Tel:65412821

Basic pathology:
Afdeling for klinisk patologi
Sygehusapotek Fyn
J.B. Winsløws Vej 15
5000 Odense C
Tel: 65411184

Gut microbiome sequencing:
Novo Nordisk fundation center for basic metabolic research
Blegdamsvej 3B
2200 Copenhagen
Tel:+4535337129
Contact: Prof. Torben Hansen
Mail:torben.hansen@sund.ku.dk

Liver expression studies:
Biomedical Research Foundation Academy of Athens
4 Soranou Ephessiou St. 115 27 Athens
Tel: +306946468849
Contact: Dr. Foteini Christodoulou
Mail:fchristodoulou@bioacademy.gr

Metabolomics:Cristina Legido Quigley

Steno Diabetes Center Copenhagen
Metabolomics Laboratory
Niels Steensens Vej 2 (NSA 2.46)
DK-2820 Gentofte
Denmark

Biomarkers:
Nordic Bioscienses A/S
Herlev Hovedgade 207
2730 Herlev
Tel: +4544525252
Contact: CEO Morten Karsdal
Mail: mk@nordicbioscience.com

Advanced Pathology
Laboratory for Fibrosis and Portal Hypertension
Department of Internal Medicine 1
University of Bonn
Sigmund-Freud-Str 25
53127 Bonn

Proteomics
Matthias Mann
mmann@biochem.mpg.de
MPI of Biochemistry, Dept. Proteomics and Signal Transduction
Am Klopferspitz 18
Martinsried, 82152

MicrobPredict
Jonel Trebicka
jonel.trebicka@efclif.com

TRAVESSERA DE GRACIA 11, 7TH FLOOR
BARCELONA 08021
Spain
Tel:4922828715507
Contact: Ass Prof Jonel Trebicka
Mail: jonel.trebicka@ukb.uni-bonn.de

Drug handling:
Sygehusapotek Fyn
J.B. Winsløws Vej 13
5000 Odense C
Tel: 6541 1184

Drug delivery:
Norgine Denmark
Stamholmen 151, 6. Floor
2650 Hvidovre
Contact: John Stone MD, Medical Manager
Tel: 33170400

Monitoring:
GCP-enheden ved OUH
Odense Universitetshospital
J.B. Winsløws Vej 19, 2 Sal
5000 Odense C
Tel: 6550 4350
Contact: Barbara Seider
bseider@health.sdu.dk

3 Sub investigators:

MD, PhD Maja Thiele
Medical student Nikolaj Torp
MD Camilla Hansen
MD Katrine Lindvig
MD Maria KjærgaardDepartment of Medical gastroenterology
Odense University Hospital
Sdr. Boulevard 29
5000 Odense C
Tel:65412755

4 Supervisors of the PhD Thesis

Main supervisor:

Professor, PhD, Aleksander Krag
Department of Medical gastroenterology
Odense University Hospital
Sdr. Boulevard 29
5000 Odense C
Tlf: +4521409915
Mail: aleksander.krag@rsyd.dk

Ass. Prof, Jonel Trebicka
Laboratory for Fibrosis and Portal
Hypertension
Department of Internal Medicine 1
University of Bonn
Sigmund-Freud-Str 25
53127 Bonn
Tel:+4922828715507
Mail: jonel.trebicka@ukb.uni-bonn.de

5 Introduction

Bacteria inhabit the human gastrointestinal tract, where they form part of the normal human physiology. Together they form a gut microbiota comprising an ecosystem with more than 2000 different species and 150-fold more genes than their human host. As most of these bacteria can´t be grown in cultures, their presence and significance has not been properly studied until the recent advancement in new DNA sequencing techniques. The study of the microbiota in health and disease is in its infancy, but an increasing body of evidence suggests that the microbiota is a co-evolved partner that significantly impact host metabolic and immunological function, and thereby is involved in human extra-intestinal disease including ischemic heart disease, diabetes and primary liver cancer (1-5). In this PhD thesis we hypothesise that the gut microbiota is a major contributor to progression of fibrosis in liver disease and that modulating gut flora by antibiotics halter disease progression. At present the only curative treatment for fibrotic end stage liver disease is liver transplantation, but this treatment is only available for a selective minority of patients. Therefore, there is a need for anti-fibrotic treatment strategies to slow down fibrogenesis, preserve liver function and reduce the burden on health care systems (6).

5.1 Microbiota induced liver fibrosis

Liver fibrosis is a wound healing response due to inflammation of the liver parenchyma following a variety of conditions such as alcohol, hepatitis B and C and non-alcoholic steatohepatitis. Prolonged inflammation stimulates extracellular matrix production from hepatic stellate cells, which accumulates and replaces the normal liver tissue. The liver is to some extend able to regenerate and reconstruct its normal architecture, however, a sustained inflammation in the liver parenchyma leads to progressive fibrosis and in the end cirrhosis (7). As intestinal blood from the large and the small intestine returns to the liver by the portal vein, the liver is the first organ which encounters bacteria and bacterial components from the gut. In normal condition, an effective gut barrier limits this inflow, and the capacity of the liver to eliminate bacterial products is not overloaded (8). In liver disease, the intestinal microbiota changes qualitatively and quantitatively in favour of a flora with increased invasive potential and the gut barrier is compromised (9). As a consequence, an increased load of bacterial products are transported to the liver where they activate Toll-like receptors, leading to pro-inflammatory gene expression and fibrogenesis (10;11). This cross talk between the intestinal microbiota and the liver constitute a gut-liver axis, which is increasingly recognized as key mechanism in the progression of liver disease and pathogenesis of liver related complications (2;12-14).

5.2 Targeting the intestinal microbiota

Modulating the microbiota by the use of broad spectrum antibiotics, is currently used in clinical hepatology at specific cirrhotic stages (15). By reducing the gram negative gut flora, antibiotics have been shown to prevent translocation of bacteria and bacterial components from the gut and thereby protect against a variety of complications in advanced liver disease (16-18). In clinical trials, antibiotic modulation of the gut flora has been proven effective in preventing spontaneous bacterial peritonitis, variceal bleeding, hepatic encephalopathy and hepatorenal syndrome (19-22).

At present there is no data evaluating a possible anti-fibrotic effect of modulating the gut flora by antibiotics in humans. However an increasing body of evidence from animal studies suggest that this is the case, including in the setting of alcoholic liver disease (23;24).

6.0 Aim and hypothesis of the study

We hypothesise that the gut microbiota and its metabolites are major drivers of fibrosis in human liver disease and that modulating the gut flora by rifaximin (a non-absorbable antibiotic) halter disease progression by intercepting pro-fibrotic influence from the gut bacteria on the liver. In specific, we hypothesise that rifaximin, compared to placebo, in this randomized controlled trial, will have the following effects.

1. Rifaximin increases the proportion of patients with an improvement in Ishak score of liver biopsies equal to or more than 1.
2. Rifaximin reduces the surrogate markers of fibrosis: collagen proportionate area, hydroxylprolin level, TIMP-1, MMP2, key pro-fibrotic cytokines (TGF-β1, PDGF-β-R, CTGF) and key pro-inflammatory cytokines (TNF-α, MCP-1) and CD 163
3. Rifaximin inhibits activation of hepatic stellate cells estimated by a reduction in the area of α-smooth muscle actin positive cells
4. Rifaximin changes the composition of the microbiota estimated by 454 pyrusequencing technology of faecal samples
5. Rifaximin changes gene expression of the microbiota estimated by transscriptomic of faecal samples
6. Rifaximin reduces host pro-inflammatory gene expression in hepatic tissue estimated by transcriptomics and microRNA profiling
7. Rifaximin affects host metabolomics of blood and urine including micro-RNA profile

Simultaneously, we want to evaluate the potential of a variety of biomarkers of fibrosis to diagnose, monitor and prognosticate the development of hepatic fibrogenesis in corporation with Nordic Bioscience.

6.1 Primary outcome: (#1)

6.2 Secondary outcomes: All others (#2-7)

7.0 Study design

Patients will be randomised 1:1 to receive rifaximin or placebo for 18 months. The study will be blinded, so neither patients nor medical staff will know whether the patient receives placebo or rifaximin.

Recruitment

From 2012 an observational study at the department of medical gastroenterology evaluated the role of the microbiota and the development of hepatic fibrosis. The inclusion criteria for this study were: a) age 18-75 years and either b) at least one year of alcohol abuse ≥24 grams/day for women and ≥36 grams/day for men. Exclusion criteria for this study are a) contraindications for liver biopsy, b) cancer or other debilitating disease with a life expectancy <1 year, c) concurrent liver disease other than alcohol, d) Human Immunodeficiency Virus, e) severe alcoholic hepatitis, f) not able to speak or read danish. Among these, a subset of 136 patients will be included in this study. The enrolment of participants in this study has stopped and consequently a new recruitment strategy is initiated from October 2019 without changing the inclusion criteria. Accordingly, the inclusion criteria from the observational study is now integrated in present study:

7.1 Inclusion criteria

1. Liver fibrosis estimated by an Ishak score from 1-4
2. Age 18-75 years and either
3. At least one year of alcohol abuse ≥24 grams/day for women and ≥36 grams/day for men.
4. Women of child-bearing potential should use safe anti-conception and provide a negative pregnancy test.

I**ntra uterine device, and non-oral hormonal contraceptives (dermal, vaginal, implant or injection) will be considered safe during trial.** According to SPC, oral hormone contraceptive will not be considered safe anti-conception. Instead use of double barrier, will be accepted**. The terminal half –life for rifaximin is 4.17 hour so patient must continue with contraceptives minimum 24 hours after last dose.**

7.2 Exclusion criteria

1. Known allergy to rifaximin
2. The investigator judge that the patient would not be compliant with trial medicine
3. Antibiotic treatment the prior 4 weeks
4. Contraindications for liver biopsy
5. Cancer or other debilitating disease with a life expectancy <1 year
6. Concurrent liver disease other than alcohol
7. Human Immunodeficiency Virus
8. Severe alcoholic hepatitis
9. Not able to speak or read Danish.

Patients suspected to have hepatic fibrosis according to liver stiffness measurement (FibroScan® >6kPa) who wants to participate and fulfil all other criteria will be offered a liver biopsy to diagnose liver fibrosis. If liver fibrosis is verified, the patient will be invited to participate and

receive written and oral information as described in section 10. If no liver fibrosis is detected no treatment is needed and the patient will be terminated without being offered participation the study.

8.0 Withdrawal from the study

1. Blinding is repealed
2. Treatment with another antibiotic for more than 4 consecutive weeks or 4 times during the study
3. The trial participant withdraws his/her written consent
4. It is considered in the participants´best interest, as judged by investigator

9.0 Dropout

Trial participants will be classified as dropouts if they meet any of the following criteria

1. The trial participant has ingested less than 75 % planned treatment
2. The trial participants do not appear to planned controls, despite contact by telephone, letter or mail

If possible, the following data on dropouts will be registered: Reason for dropout, mortality, amount of ingested trial medicine, all patient data collected during the trial.

10 Recruitment and enrolment of participants

Physician investigators associated to ongoing research projects on alcohol related liver disease at Liver Research Centre will identify patients eligible for trial. Patient will receive oral information about the trial and be offered inclusion. Trial information and written consent is handed out to the patient. Subsequently the patient will sign the informed consent if they accept to participate in the trial.

11 Time schedule:

1/9-2014 Enrolment of first patient and start of investigations
31/07-2020 Enrolment of last patient
31/12-2021 End of follow up, trial shut-down
31/12-2031End of analysis and data assessment

12 Drug information

Rifaximin is a semi-synthetic analogue of the antimicrobial rifampicin. Oral administration of rifaximin results in very high faecal concentrations and less than 0,4 % is absorbed. Rifaximin acts by inhibiting RNA synthesis and possesses a broad spectrum of activity against Gram-positive and

–negative bacteria both aerobic and anaerobic. In contrast to other antibiotics, rifaximin does not appear to lead to bacterial resistance (25). Rifaximin is approved by the FDA (U.S. Food and Drug administration) for the reduction of recurrence of overt hepatic encephalopathy at a dosage of 550 mg twice a day (21). In Denmark rifaximin is approved for the treatment of both travellers´ diarrhoea caused by non-invasive gut bacteria and prevention of recurrence of hepatic encephalopathy. The recommended dosage is 200 mg thrice daily for three days for travellers´ diarrhoea and 550 mg twice a day in hepatic encephalopathy(26;27). We have chosen to modulate the gut microbiota by rifaximin as it has an excellent safety profile, specifically addresses the gut flora and data suggest that it may lead to less bacterial resistance(25). Other antibiotics such as quinolones currently used in clinical hepatology have unintended effects on the extra-intestinal bacterial flora and their long term use is associated with the development of bacterial resistance(28;29).

To secure effective modulation of the gut flora we have chosen the increased dosage of 550 mg twice daily, as used in the prevention of hepatic encephalopathy (21;27). This dosage has been shown to reduce translocation of bacterial products, the key mechanism in the pro-fibrotic gut-liver cross talk (30). As hepatic fibrogenesis is a slow evolving process, we expect a necessary 18 months of treatment period to be able to detect changes (31). Rifaximin as well as placebo will be delivered from Norgine Denmarc free of charge. Placebo tablets will be similar in size, shape and weight as the rifaximin tablet.

13 Side effects, risks and disadvantages related to medication

The tolerability of long-term use of rifaximin in patients with liver cirrhosis has been evaluated in a clinical phase 3 trial involving 299 patients. The patients were randomized to placebo (159) or rifaximin (140) 550 mg twice a day for 6 month in the setting of preventing recurrent hepatic encephalopathy. Rifaximin significantly reduced the risk of a break through episode of hepatic encephalopathy from 46 % (placebo) to 22 % among patients receiving rifaximin. The majority of reported side effects from this study were of gastrointestinal origin as nausea and abdominal discomfort. The incidence of adverse events and serious adverse events were similar in patients receiving placebo or rifaximin (21). Possible side effects of rifaximin according to the summary of product characteristic (SPC) are listed below.

**MedDRA-systemorgan-klasse**	**Almindelig**	**Ikke almindelig**	**Sjælden**	**Ikke kendt**
**Infektioner og parasitære sygdomme**		Clostridium-infektion, urinvejsinfektion, candidiasis	Lungebetændelse, cellulitis, infektion i de øvre luftveje, rhinitis	
**Blod og lymfesystem**		Anæmi		Trombocytopeni
**Metabolisme og ernæring**		Anoreksi, hyperkaliæmi	Dehydrering	

MedDRA-systemorgan-klasse	Almindelig	Ikke almindelig	Sjælden	Ikke kendt
**Psykiske forstyrrelser**	Depression	Konfusion, angst, hypersomnia, søvnløshed		
**Nervesystemet**	Svimmelhed, hovedpine	Balanceforstyrrelser, amnesi, kramper, opmærksomhedsforstyrrelser, hypoæstesi, hukommelsessvigt		Anafylaktiske reakioner, angioødem, overfølsomhed
**Vaskulære sygdomme**		Hedeture	Hypertension, hypotension	Præsynkope, synkope
**Luftveje, thorax og mediastinum**	Dyspnø	Pleuraeffusion	Kronisk obstruktiv lungesygdom	
**Mave-tarm-kanalen**	Smerter i den øvre del af maven, oppustethed, diarré, kvalme, opkastning, ascites	Mavesmerter, øsofageale varicer, blødninger, mundtørhed, maveubehag	Forstoppelse	
**Lever og galdeveje**				Anomale leverfunktionstest
**Hud og subkutane væv**	Udslæt, pruritus			Dermatitis, eksem
**Knogler, led, muskler og bindevæv**	Muskelspasmer, artralgi	Myalgi	Rygsmerter	
**Nyrer og urinveje**		Dysuri, pollakisuri	Proteinuri	
**Almene symptomer og reaktioner på administrations-stedet**	Perifert ødem	Ødem, pyreksi	Asteni	
**Undersøgelser**				Anomale INR-værdier
**Traumer, forgiftninger og behandlings-**		Fald	Blå mærker, proceduresmerter	

MedDRA-systemorgan-klasse	Almindelig	Ikke almindelig	Sjælden	Ikke kendt
komplikationer				

Hyppigheden defineres som følger: Meget almindelig (≥1/10), almindelig (≥1/100 til <1/10), ikke almindelig (≥1/1.000 til <1/100), sjælden (≥1/10.000 til <1/1.000), meget sjælden (<1/10.000), ikke kendt (kan ikke estimeres ud fra forhåndenværende data)

14 Possible benefits of rifaximin for trial participants

Rifaximin has been shown to reduce disease severity, lower portal pressure and ameliorate the deranged systemic haemodynamic state seen in cirrhosis (30;32;33). In selected cases, long-term use of rifaximin may improve survival and protect against complications as hepatorenal syndrome, spontaneous bacterial peritonitis, variceal bleeding and encephalopathy (34). As the patients included in this trial are in an early stage of liver disease, it is less likely that they will experience these benefits. However, we expect that rifaximin halter disease progression by limiting hepatic fibrogenesis and potentially improve the outcome for trial participants treated with rifaximin.

15 Handling of trial medication

The patients will receive one tablet containing 550 mg of rifaximin twice daily for 18 months in total. The patients randomized to placebo will receive one tablet of placebo twice daily for 18 months. Trial medication is an oral tablet that can be ingested with a glass of water. Rifaximin tablets and placebo will be similar in size, colour, shape and form. The dosage is two times daily, morning and evening. If patients forget to ingest the morning or evening tablet, they may take the missing dose along with the next tablet. The pharmacy will label medication and placebo bags for each participant. One back will comprise 10 blisters of 14 tablets equivalent of two months of trial medication (140 tablets). The pharmacy will label medication and placebo according to GMP guidelines for labelling of medication for clinical trials. Due to work flow and weekends it is not possible to guarantee liver biopsy and follow up. A window of 12 weeks is therefore allowed in the trial

16 Record keeping of trial medication

Investigators or a study nurse will keep record of medication and hand out new trial medication to the patients in the outpatient clinic at the months 1,2,4,6,8,10,12,14,16,18. Used and un-used medication will be registered to keep track of the ingested amount for each participant. The registration includes amount of medicine delivered from the pharmacy, amount of medicine handed out to patients and amount of unused medication returned by the participant. Patients

will be informed both orally and in writing to return all unused mediation to the hospital and investigators. The participant will be asked to keep a journal of medicine intake. In case of uncertainty regarding the amount of ingested medicine, the highest certain dose will be registered. If participants are unable to account for medicine or trial medication is lost, the ingested dose will be registered as null.

17 Randomisation methods

The study is conducted as a randomized placebo controlled clinical trial. Patients will be randomized to receive rifaximin or placebo in a ratio of 1:1. Randomization will be performed in blocks of 4 and will be stratified according to the initial metavir score and whether or not participants are abstinent the previous 6 months. The study is dimensioned to include 136 patients based on a power calculation. Packing and labelling will take place the hospital pharmacy. Labelling will be performed according to legislation including name of pharmacy, trial name and trial number, dose, storage and contact information of principle investigator. The randomization list will be generated electronically by a central computer and stored at the pharmacy and only personnel working there will know the code. The sponsor, investigators, nurses, laboratory assistants or personnel involved in the care of trial participant will have no knowledge of the randomization. After randomization, a three digit trial number will henceforth identify trial participants. Coded envelopes with randomisation keys to each participant will be kept at Odense University Hospital. The sponsor and investigators have aces to the coded envelopes at all times if un-blinding should be necessary.

18 Disruption of blinding

Blinding will be uncovered in case of any of the following events should occur:

1. A trial participant experiences serious adverse side effects or complications that could be caused by rifaximin
2. A trial participant is admitted to hospital with a severe or life-threatening condition in which treatment with rifaximin is contraindicated

Coded envelopes containing allocated treatment (rifaximin or placebo) for each participant will be stored at the drug depository at the outpatient clinic. Nursing staff from the department has access to the drug depository 24 hours a day by using a unique code. Thus, the attending physician can disclosure allocated treatment for a trial participant, if judged necessary, at anytime. If blinding is disclosed, the attending physician will inform primary investigator or sponsor by telephone next working day. This will be recorded in the journal. Registration of adverse event/ adverse reaction will be registered as described in chapter 20.
The patient will be excluded from the trial, if blinding is disclosed.

19 Adverse events and side effects

The following definitions regard to medical treatment, investigations and invasive procedures performed during this trial.
An adverse event is: Any untoward medical occurrence in a patient or clinical trial subject administered a medicinal product and which does not necessarily have a causal relationship with this treatment.
A serious adverse event is: Any untoward medical occurrence or effect that at any dose results in death, is life-threatening, requires hospitalisation or prolongation of existing hospitalisation, results in persistent or significant disability or incapacity, or is a congenital anomaly or birth defect.
An adverse reaction is: Any harmful and unwanted reaction toward a trial drug irrespectively of dose
An unexpected adverse reaction is: Any harmful and unwanted reaction toward a trial drug, whose character or severity does not correlate to product information (SPC). The SPC for Xifaxan as specified in section 13 will be used to evaluate if an adverse reaction is expected or unexpected

Events and reactions that will not be handled as serious adverse events:
-Adverse events and reactions that with certainty are occurring in relation to or are caused by the treatment and diagnosis of other medical conditions or diseases besides liver disease during trial period will be registered but not reported.
-Abnormal blood analyses commonly seen in patients with alcoholic or advanced fibrotic liver disease such as elevated liver enzymes, low platelet count or albumin, increase in INR or P-bilirubin.
In any questions of doubt regarding the above, investigators will inform sponsor that will assess possible causality.
-Hospitalisations in relation to performing the liver biopsy due to the need of observing patients beyond normal opening hours in the outpatient clinic.

20 Registration of adverse event/adverse reaction

Investigators and sponsors are obliged to comply with protocol including reporting all adverse events, serious adverse events and suspected unexpected serious adverse reaction to relevant authorities as outlined by the Danish Health and Medicine Authority and The European Commission (35;36). During the planned follow up, trial participant will be asked if an adverse event has occurred since the last contact. All adverse events are registered in the Case Report Form. Serious adverse events are also registered on the SAE form included in Trial Master File. All adverse events will be followed until stabilisation or termination. After terminating the study, a final report of registered reactions and events in the CRF will be send to The Danish Health and Medicines Agency and Ethics committee.

20.1 Reporting of Adverse Events (AE)

Adverse events/reactions, that are not life threatening and mild in character, do not require medical attention or intervention and are insignificant to the patients´ further involvement in this trial will not be reported to sponsor. These will be registered in case report forms and available to monitoring units and sponsor at any time.

20.2 Reporting of Serious Adverse Events (SAE)

Serious adverse events is reported to the principle investigator (Mads Israelsen) or sponsor (Aleksander Krag) no later than the first workday after investigators have become aware of the event. Investigators will fill out the form ”skema til indberetning af SAE/SUSAR” available in the in trial master form and put it in the sponsors mailbox. The sponsor will additionally be informed orally. If either of the investigator or sponsor judge that the serious adverse event is related to trial medication (a serious adverse reaction), the sponsor will assess whether the serious adverse reaction is expected according to the SPC for Xifaxan in section 13 (27). If expected it will be considered a serious adverse reaction, if unexpected (not previously described in the product resume) it will be considered a Suspected Unexpected Serious Adverse Reaction (SUSAR). SAE and SAR will be reported yearly in a safety report to both The Danish Health and Medicines Agency and the Ethics committee.

20.3 Reporting of Suspected Unexpected Serious Adverse Reaction (SUSAR)

In case of death or a life threatening condition, the SUSARS will be reported by the sponsor to the The Danish Health and Medicines Agency within 7 days of the sponsors´ knowledge of the reaction. Within 8 days the sponsor and the principal investigator will send a report of the follow up of the incident. All other SUSAR´s will be reported within 15 workdays. Reporting will be done electronically. In case of a SUSAR, un-blinding will be performed before reporting to the authorities. SUSAR´s will also be reported to the Etics committee as described above.

21 Measurements and investigations

Scanning of the liver with fibroscan and Explorer will be done according to Standard Operational Procedures described in appendix 1 & 2. The remaining scheduled investigations in this trial are standard procedures at the department of medical gastroenterology. Blood analyses are standard procedures at the department of clinical biochemistry. Standard Operating Procedures for investigations are therefore available to investigators, nurses and other personnel on the intranet for Odense University Hospital.

Investigations day 0

The investigator registers the patient, verifies written consent and supplements information about the trial if needed. Measurement and investigations from the study (including liver biopsy) “Forekomst af leversygdom hos patienter med aktuelt eller tidligere alcohol overforbrug” project-ID S-20120071 constitute the baseline values and will not be repeated at inclusion. This however

does not include samples from urine, fecal, saliva and blood which will be repeated at inclusion and these samples may constitute baseline values if judged appropriate by the sponsors in case of a prolonged time span between the two studies.

- Urine HCG to exclude pregnancy in women of childbearing age
- Randomization to placebo or rifaximin
- Oral and written information about handling of study medication
- Study medication is handed out to patient

The baseline values from project ID S-20120071 constitute of: a) liver biopsy, b) venous fasting blood, c) feces, d) urine, e) saliva. Non-invasive assessments of fibrosis are performed using transient elastography, real-time quantitative shear wave elastography and the latest serological markers. Nutritional status is evaluated by combining hand-grip-strength, mid-upper-arm circumference and body mass index. Health-related quality of life is measured using generic (SF-36) and disease specific questionnaires (CLDQ). Alcohol dependency is evaluated using the CAGE questionnaire. Abdominal ultrasonography is performed in all patients

Investigations 1 month

Patients will be seen by an investigator or study nurse in the outpatient clinic

- Tablet ingestion and compliance
- Adverse Events, and complications in case report form
- Blood samples including ELF and standard blood samples
- Urine, sputum, faecal and hair samples (ethyl glucoronide)
- Study medication is handed out to patients

Investigations 2,4,6,8,10,12,14,16 Month

Patients will be seen by an investigator or study nurse in the clinic

- Tablet ingestion and compliance
- Adverse Events, and complications in case report form (CRF)
- Study medication is handed out to patients
- Registration of alcohol consumption by interview
- Blood samples/test?

Investigations 18 Month

Patients will be seen by an investigator or study nurse in the clinic

- Tablet ingestion and compliance
- Adverse Events and complications in case report form

- Liver biopsy
- Standard blood samples, including venous fasting blood
- Non-invasive assessments of fibrosis are performed using transient elastography, real-time quantitative shear wave elastography and the latest serological markers including ELF
- Feces
- Urine
- Saliva
- Nutritional status is evaluated by combining hand-grip-strength, mid-upper-arm circumference and body mass index.
- Health-related quality of life is measured using generic (SF-36) and disease specific questionnaires (CLDQ)
- Alcohol dependency is evaluated using the CAGE questionnaire and measurement of ethyl glucoronide in hair samples
- Abdominal Ultrasound

Optional Follow up meeting 19 Month

Patients will be seen by an investigator or regular physician

The Patient will be informed about the result of the liver biopsy.

22 Risks and disadvantages related to investigations and measurements

Percutaneous liver biopsy is considered a safe investigation with few complications in the setting of diagnosing non-malignant hepatic disorders. However, most patients experience some pain related to the site of the puncture. Potential risks related to the procedure are; bleeding, bile peritonitis and perforation of the gallbladder. The mortality is 1/10.000, however no deaths have been directly related to the procedure when performed in the setting of diagnosing abnormal liver function tests. Major bleeding episodes (the most frequent complication), appear in 2,2 per 1000 biopsies in patients investigated for abnormal liver function test (37). To minimize risk for the trial participants, the procedure will only be done by the trial investigators or medical staff with extensive experience. Liver biopsy is a routine procedure in the department of medical gastroenterology and the department has the setup to handle complications if they should occur. There is no discomfort or risk related to ultrasound or elastiometric done by the Supersonic Aixplorer or Fibroscan-touch.

23 Establishment of a Bio bank

In connection to this study a research bio bank and bio bank for future research will be established. The objective is that the bio banks will contain material from trial participants before and after trial medication. The material is full blood, EDTA blood, urine, faeces, sputum, hair and hepatic tissue. The blood, urine, faeces, sputum, hair and hepatic tissue will be stored at a

temperature of – 80 degrees Celsius. The establishment of the two bio banks will serve two different purposes. The research bio bank will contain the samples that for various reasons are not are ready for immediate analysis. These samples will be stored in a research bio bank during the trial period. This accounts for blood, urine faeces, sputum, hair and hepatic tissue. Data from these samples will enter the database and registers of the trial and excess material from these samples will be destroyed. All material is personally identifiable. The primary investigator will store results of analyses until statistical analysis. Storage of data, trial material and case report forms will be kept in accordance to general guidelines for storage of personally identifiable material, set by The Danish Data Protection Agency. A bio bank for future research will be established, the aim is to contribute to improvement in the treatment of liver disease. This bio bank will contain blood, urine, faeces, sputum, hair and hepatic tissue. The material is personally identifiable. As the technical know-how of some planned analysis is not available in Denmark, there is a need to transfer material from the research bio bank to international research-partners. The bio bank for future research is not directly related to the current project but will be registered at the Danish Data Protection Agency. When the bio bank is needed, permission will be obtained at the Regional Science Ethics Committee and Data Protection Agency before new research is started. The material in the bio bank will be stored for a maximum of 30 years and thereafter be destroyed.

24 Data management

Data in the trial is obtained from the patients´ oral statements, medical records, laboratory results and investigational results. Basic demographic data and prior medical history of trial participants is obtained from the study “Forekomsten af leversygdom hos mennesker med aktuelt eller tidligere alkohol overforbrug” project-ID S-20120071.Data will be stored and kept in the electronic case report form, which functions as an encrypted database. All data is handled confidentially. The three-digit number and CPR will identify patients. Investigational and laboratory results are manually transferred to the electronic CRF by investigators or a study nurse. Only staff handling the care and registration of trial participant located at Odense University Hospital will have encoded access to the electronic CRF. The electronic CRF is accessed electronically with personal user ID and password. The primary investigator will extract data at the end of the inclusion period. The primary analysis addresses the primary outcome of the trial, the secondary analysis addresses secondary outcomes. It is the intention to publish all results obtained from this trial. The data will be published in an anonymous form. In the case of missing data; Investigators repeat non invasive procedures including blood samples when it is possible to complete data. If the parameter is not available at the time of the trial analysis, the registration will be let empty. Invasive procedures (liver biopsy) will not be repeated if data is missing

25 Statistics

Liver fibrosis due to alcoholic abuse regress in 27 % of patients, is unchanged in 57 % and progress in 16 % of patients in a 2 year period, if patients with hepatitis C are excluded. The high rate of regression is due to the fact that many patients with alcohol abuse stop drinking or reduce their alcohol consumption when included in clinical trials (38). We therefore expect that 14 % will regress during a 1 year period. The smallest relevant difference is considered a 25 % absolute increase in the percentage of patients who regress 1 or more in their histological score. If setting

the risk of performing a type 1 error to 5% and a type 2 error to 20 %, 136 patients are needed when performing a power calculation. This includes a dropout rate of 20 %. Both intention to treat and per protocol analysis will be performed.

26.0 Ethics

The study will be conducted according to the national legislation, the ICH-GCP guidelines and the protocol. The GCP unit at Odense University Hospital will monitor the trial according to GCP guidelines. Permission from the Regional Science Ethics Committee of Southern Denmark, The Danish Data Protection Agency and Danish Health and Medicine Agency will be obtained before initiation of the trial. The GCP unit and authorities will have full access to data, documents and registration procedures during monitoring, audits and inspections.
During the study we will extract the total RNA from the liver biopsies to do whole transcriptome studies (for in depth description see appendix 10) and perform whole genomic sequencing. The project will only focus on identification of alcoholic liver disease causing mutations, and not on identification of variants associated with other phenotypes. Participants will only be contacted regarding identification of putative disease causing mutations after a new approval from The Regional Ethical Committee of Southern Denmark. In the event of coincidence findings in this research project, in the form of a genetic variant, which is known to cause a disease for which there is a treatment and / or prevention ability of the subject and his family members which may carry the same mutation, the genetic finding will be verified by the Sanger sequencing. Provided that the mutation is confirmed and known to cause disease with significant penetrance, we will set up a local genetics ethics panel consisting of a molecular genetics, a specialist in clinical genetics with experience in genetic counseling and a specialist in the specific disease, which is triggered by the mutation, in order to draw up an action and communication plan with respect to the mutation carrier. If the panel is in doubt of the action plan, the Regional Ethical Committee of Southern Denmark will be contacted. If it is concluded that there are medical reasons (see above) to notify the genetic findings to the subject, the dissemination will be performed by a doctor related to the project under the instructions of the panel specialist in clinical genetics and specialist in the specific disease.

26.1 Obtaining consent

To avoid the risks from taking a baseline liver biopsy and minimize inconvenience for trial participants, we will only recruit participant who has already fulfilled the on-going research project “Forekomsten af leversygdom hos mennesker med aktuelt eller tidligere alkohol overforbrug”. Measurements and investigations from this research project (which includes a liver biopsy) will serve as baseline values before treatment with trial medication. The project “Forekomsten af leversygdom hos mennesker med aktuelt eller tidligere alkohol overforbrug” ID S-20120071 is approved by the Regional Science Ethics Committee of Southern Denmark, The Danish Data Protection Agency and has presently included 140 of the planned 400 patients. The participants of this project will be informed about the existence of this clinical trial by the investigators or their regular physician as they come to planned follow up in the ambulatory. If interested in participating in this project, the patients will be informed orally and receive written participant information. This includes the writings “Forsøgspersoners rettigheder i et sundhedsvidenskabligt forskningsprojekt” and “Deltagerinformation”. The patient will then be booked for inclusion with

the one of the trial investigators. The patient will be informed that he or she is entitled to a minimum of 24 hours of reflection, can request an extra interview where he or she can be orally informed, and that he or she can bring a counselor. This will be done in a quiet room in the out-patient clinic. Contact information for the principle investigator will be provided in oral and writing. The investigator will secure that the patient has read and understood the written participant information and will afterward be informed in oral. The patient can subsequently chose to sign the written consent form. When written consent is obtained, the patient is enrolled and the consent is forwarded to principle investigator. The few patients who has already participated in the The project “Forekomsten af leversygdom hos mennesker med aktuelt eller tidligere alkohol overforbrug” ID S-20120071 and doesn´t have a scheduled follow up in the ambulatory, will be invited to participate in the current study by postmail. The postmail will contain the patient information and an invitation to participate the study. Patient will be informed that they should contact the investigators by telephone or mail if they wish to participate in the trial. This procedure for information and obtaining consent is chosen to avoid contacting patients further.

26.2 Patient disadvantages and risk

Participants randomized to rifaximin may experience side effects to this treatment as elaborated in chapter 10. Risks related to investigations are elaborated in chapter 19. Participation in this trial will require that the patient after randomization will be seen at follow up after 1,2,4,6,8,10,12,14,16,18 months. Investigations at the 18 month will require that the patient set of one day, due to the need for observation after conducting the liver biopsy and repeating of sonography. The remaining consultations are estimated to last 15 minutes each. Half of all patients will receive placebo medicine. Currently there is no known effective medicine to treat fibrotic liver disease and it lacks to be proven that rifaximin has an anti-fibrotic effect. Hence, no patient will be withheld proved active medication by participating in this trial. All patients continue their regular prescribed medication during this trial. Patients receiving placebo are unlikely to experience any improvements in their disease or symptoms. However it is necessarily to treat half of patients with placebo, to be able to ascribe observed effects to rifaximin with certainty.

26.3 Patient Benefit

Participation in this trial will contribute to new knowledge, enabling us to improve diagnosis and treatment of fibrotic liver disease. It is possible (but not certain) that patients receiving rifaximin experience improvement in their liver disease. It is likely, that patients with advanced fibrotic hepatic disease have fewer liver related complications when treated with rifaximin. Overall, we have evaluated that the benefits of participating in this trial outnumbers the risks and we find the trial ethically safe.

27 Insuring and Financing

The national patient insurance will insure patients participating in this trial. Sponsor and investigators are covered by Odense University Hospital´s statutory insurance. Norgine Denmark is

covered by their own product liability insurance which covers faults in medicine. Rifaximin tablets of 550 mg in the amount of 78 blisters of 14 tablets for 68 participants and placebo tablets 78 blister of 14 tablets for 68 participants are sponsored from Norgine Denmark free of cost. The medicine is sponsored unconditionally. That is, Norgine Denmark has no influence in designing, management or reporting of the trial. Norgine Denmark has not provided additional funding and is not further involved in the trial. The sponsor and the principal investigator have started the project and designed the trial. None of the involved researchers have economic interests or affiliations in private firms or funds. The study has gained support from Højteknologi fonden as it involves cooperation with a private company Nordic bioscience. Nordic bioscience has the patent of Protein Fingerprinting and associated markers of fibrosis and thereby has economically interest in the study. Selective participants can have their travel-costs reimbursed. Patient will be reimbursed according to actual cost (documented by receipt) or in case they travel in their own car, they will be reimbursed according to driving subsidies in the Danish state 3.53 Kr./km. The study has obtained financial support from Horizon 2020, EU's programme for science and innovation under the fund number 668031.

Additional funding during the study will be reported to the Regional Science Ethics Committee of Southern Denmark and in the "skriftlige deltagerinformation" which is handed out to trial participants.

**Budget**	**Finansiering**	**Beløb**	
**Lønninger**			
1. års VIP løn inkl. Studieafgift	Søges ved Region Syddanmarks PhD. Pulje	573.655	DKK
2. års VIP løn inkl. Studieafgift	Bevilliget af højteknologi fonden	573.655	DKK
3. års VIP løn inkl. Studieafgift	Bevilliget af højteknologi fonden	573.655	DKK
**Apparatur**			
Fibroskanner	Bevilliget via Odense Universites hospitals Frie Forskningsmidler	635.000	DKK
It-udstyr	Søges ved Syddansk Universitets Forskningsfond	17.000	DKK
Supersonic Aixplorer	Bevilliget af AP Møller- og Toyotafonden	935.000	DKK
**Drift**			
**Afdelingen for klinisk biokemi og farmakologi ved Odense Universitets Hospital:**			
Udgifter til biokemiske analyser	Søges ved Fionia fonden	150.000	DKK
**Københavns Universitets Metabolisme center:**			
Undersøgelse af tarmfloraen	80.000 DKK er bevilliget via Knud og Edith Eriksens Mindefond.	1.020.000	DKK
	Søges ved eksterne fonde	250.000	DKK

**Biomedical Research Foundation, Athens:** Undersøgelse af levervæv			
**VTT Technical Research centre of Finland:** Undersøgelse af stofskiftet	Søges ved eksterne fonde	150.000	DKK
Øvrig drift	Bevilliget via Odense Universitets Hospitals frie forskningsmidler (Via GALAXY ansøgning)	90.000	DKK
**Totale udgifter**		**4.967.965**	**DKK**
**Financeret**		**3.152.310**	**DKK**
**Manglende finansiering**		**1.815.655**	**DKK**

28 Publication

The trial will be registered in www.clinicaltrials.gov. Both negative and positive results will be published. It is expected that the trial will lead to several publications in leading journal in the field of Hepatology. Primary investigator Mads Israelsen will be primary author on major publication regarding clinical aspects of this trial. Sponsor and participating investigators will be co-authors according to their work and involvement in the trial. If the participant has chosen to be informed about the results of the trial, they will receive a writing about the result of the trial and whether the participant has received rifaximin or placebo.

29 Implications

The trial in this PhD thesis is a proof of concept study that elucidates the role of pro-fibrotic cross talk between gut bacteria and the liver. If gut bacteria are significant contributors to liver fibrosis, modulation of the gut flora by antibiotics, probiotics, fecal transplantation and other treatment options could be a novel way to improve outcome in liver disease (14). This PhD thesis will involve collaboration with international research partners, leading to inflow of know-how to the Danish research community and the consolidation of a state of the art research platform in the department of medical gastroenterology in Odense.

Reference List

(1) Hooper LV, Littman DR, Macpherson AJ. Interactions between the microbiota and the immune system. Science 2012 Jun 8;336(6086):1268-73.

(2) Chassaing B, Etienne-Mesmin L, Gewirtz AT. Microbiota-liver axis in hepatic disease. Hepatology 2013 May 22.

(3) Tang WH, Wang Z, Levison BS, Koeth RA, Britt EB, Fu X, et al. Intestinal microbial metabolism of phosphatidylcholine and cardiovascular risk. N Engl J Med 2013 Apr 25;368(17):1575-84.

(4) Vrieze A, van NE, Holleman F, Salojarvi J, Kootte RS, Bartelsman JF, et al. Transfer of intestinal microbiota from lean donors increases insulin sensitivity in individuals with metabolic syndrome. Gastroenterology 2012 Oct;143(4):913-6.

(5) Yoshimoto S, Loo TM, Atarashi K, Kanda H, Sato S, Oyadomari S, et al. Obesity-induced gut microbial metabolite promotes liver cancer through senescence secretome. Nature 2013 Jul 4;499(7456):97-101.

(6) Blachier M, Leleu H, Peck-Radosavljevic M, Valla DC, Roudot-Thoraval F. The burden of liver disease in Europe: a review of available epidemiological data. J Hepatol 2013 Mar;58(3):593-608.

(7) Fujii H, Kawada N. Inflammation and fibrogenesis in steatohepatitis. J Gastroenterol 2012 Mar;47(3):215-25.

(8) Nolan JP. The role of intestinal endotoxin in liver injury: a long and evolving history. Hepatology 2010 Nov;52(5):1829-35.

(9) Wiest R, Garcia-Tsao G. Bacterial translocation (BT) in cirrhosis. Hepatology 2005 Mar;41(3):422-33.

(10) Seki E, Schnabl B. Role of innate immunity and the microbiota in liver fibrosis: crosstalk between the liver and gut. J Physiol 2012 Feb 1;590(Pt 3):447-58.

(11) Yang L, Seki E. Toll-like receptors in liver fibrosis: cellular crosstalk and mechanisms. Front Physiol 2012;3:138.

(12) Szabo G, Bala S, Petrasek J, Gattu A. Gut-liver axis and sensing microbes. Dig Dis 2010;28(6):737-44.

(13) Seo YS, Shah VH. The role of gut-liver axis in the pathogenesis of liver cirrhosis and portal hypertension. Clin Mol Hepatol 2012 Dec;18(4):337-46.

(14) Madsen BS, Havelund T, Krag A. Targeting the Gut-Liver Axis in Cirrhosis: Antibiotics and Non-Selective beta-Blockers. Adv Ther 2013 Jul 24.

(15) EASL clinical practice guidelines on the management of ascites, spontaneous bacterial peritonitis, and hepatorenal syndrome in cirrhosis. J Hepatol 2010 Sep;53(3):397-417.

(16) Cirera I, Bauer TM, Navasa M, Vila J, Grande L, Taura P, et al. Bacterial translocation of enteric organisms in patients with cirrhosis. J Hepatol 2001 Jan;34(1):32-7.

(17) Albillos A, de la Hera A, Gonzalez M, Moya JL, Calleja JL, Monserrat J, et al. Increased lipopolysaccharide binding protein in cirrhotic patients with marked immune and hemodynamic derangement. Hepatology 2003 Jan;37(1):208-17.

(18) Zapater P, Frances R, Gonzalez-Navajas JM, de la Hoz MA, Moreu R, Pascual S, et al. Serum and ascitic fluid bacterial DNA: a new independent prognostic factor in noninfected patients with cirrhosis. Hepatology 2008 Dec;48(6):1924-31.

(19) Gines P, Rimola A, Planas R, Vargas V, Marco F, Almela M, et al. Norfloxacin prevents spontaneous bacterial peritonitis recurrence in cirrhosis: results of a double-blind, placebo-controlled trial. Hepatology 1990 Oct;12(4 Pt 1):716-24.

(20) Hou MC, Lin HC, Liu TT, Kuo BI, Lee FY, Chang FY, et al. Antibiotic prophylaxis after endoscopic therapy prevents rebleeding in acute variceal hemorrhage: a randomized trial. Hepatology 2004 Mar;39(3):746-53.

(21) Bass NM, Mullen KD, Sanyal A, Poordad F, Neff G, Leevy CB, et al. Rifaximin treatment in hepatic encephalopathy. N Engl J Med 2010 Mar 25;362(12):1071-81.

(22) Fernandez J, Navasa M, Planas R, Montoliu S, Monfort D, Soriano G, et al. Primary prophylaxis of spontaneous bacterial peritonitis delays hepatorenal syndrome and improves survival in cirrhosis. Gastroenterology 2007 Sep;133(3):818-24.

(23) Zhu Q, Zou L, Jagavelu K, Simonetto DA, Huebert RC, Jiang ZD, et al. Intestinal decontamination inhibits TLR4 dependent fibronectin-mediated cross-talk between stellate cells and endothelial cells in liver fibrosis in mice. J Hepatol 2012 Apr;56(4):893-9.

(24) Adachi Y, Moore LE, Bradford BU, Gao W, Thurman RG. Antibiotics prevent liver injury in rats following long-term exposure to ethanol. Gastroenterology 1995 Jan;108(1):218-24.

(25) Gerard L, Garey KW, DuPont HL. Rifaximin: a nonabsorbable rifamycin antibiotic for use in nonsystemic gastrointestinal infections. Expert Rev Anti Infect Ther 2005 Apr;3(2):201-11.

(26) Produkt resume Xifaxan 200 mg. Lægemiddelstyrelsen . 2011.
Ref Type: Magazine Article

(27) Produkt resume Xifaxan 550 mg. Sundhedsstyrelsen . 2013.
Ref Type: Magazine Article

(28) Fernandez J, Navasa M, Gomez J, Colmenero J, Vila J, Arroyo V, et al. Bacterial infections in cirrhosis: epidemiological changes with invasive procedures and norfloxacin prophylaxis. Hepatology 2002 Jan;35(1):140-8.

(29) Aparicio JR, Such J, Pascual S, Arroyo A, Plazas J, Girona E, et al. Development of quinolone-resistant strains of Escherichia coli in stools of patients with cirrhosis undergoing norfloxacin prophylaxis: clinical consequences. J Hepatol 1999 Aug;31(2):277-83.

(30) Vlachogiannakos J, Saveriadis AS, Viazis N, Theodoropoulos I, Foudoulis K, Manolakopoulos S, et al. Intestinal decontamination improves liver haemodynamics in patients with alcohol-related decompensated cirrhosis. Aliment Pharmacol Ther 2009 May 1;29(9):992-9.

(31) Schuppan D, Afdhal NH. Liver cirrhosis. Lancet 2008 Mar 8;371(9615):838-51.

(32) Kalambokis GN, Tsianos EV. Rifaximin reduces endotoxemia and improves liver function and disease severity in patients with decompensated cirrhosis. Hepatology 2012 Feb;55(2):655-6.

(33) Kalambokis GN, Mouzaki A, Rodi M, Pappas K, Fotopoulos A, Xourgia X, et al. Rifaximin improves systemic hemodynamics and renal function in patients with alcohol-related cirrhosis and ascites. Clin Gastroenterol Hepatol 2012 Jul;10(7):815-8.

(34) Vlachogiannakos J, Viazis N, Vasianopoulou P, Vafiadis I, Karamanolis DG, Ladas SD. Long-term administration of rifaximin improves the prognosis of patients with decompensated alcoholic cirrhosis. J Gastroenterol Hepatol 2013 Mar;28(3):450-5.

(35) Vejledning til ansøgning om tilladelse til kliniske forsøg med lægemidler på mennesker. Sundhedsstyrelsen 2013.

(36) Detailed guidance on the collection, verification and presentation of adverse event/reaction report arising from clinical trials on medical products for human use. Official Journal of the European Union 2013.

(37) West J, Card TR. Reduced mortality rates following elective percutaneous liver biopsies. Gastroenterology 2010 Oct;139(4):1230-7.

(38) Lieber CS, Weiss DG, Groszmann R, Paronetto F, Schenker S. II. Veterans Affairs Cooperative Study of polyenylphosphatidylcholine in alcoholic liver disease. Alcohol Clin Exp Res 2003 Nov;27(11):1765-72.

Appendix 1:Standard operational procedures for shear wave elastography with *Supersonic Aixplorer.*

1. The patient must be fasting form midnight, however a glass of water is allowed until 2 hours prior to the examination.
2. In the liver, SWE measurements are performed on the right lobe, through intercostal spaces with the patient lying in the supine position with the right arm in maximal abduction. The same intercostal space should preferably be used for SWE measurements and liver biopsy. The biopsy is done after SWE

3. Use SWE box size of 3.5 - 2.5 cm.
4. The upper edge of the SWE box are placed 1.5-2.0 cm from Glisson's capsule in the liver and in an area of parenchyma free of large vessels.
5. Measurements of liver stiffness are obtained from the average of a circular ROI (≥15mm in diameter), when permitted by scanning conditions.
6. The circular ROI can be reduced in diameter, if limitations in viable signal within the SWE box prohibits a 15mm diameter.
7. Measurements are classified as failed when no or little signal can be obtained in the SWE box for all acquisitions or if the standard deviation exceeds 30% of the mean.
8. Three consecutive measurements are obtained.
9. In the spleen, SWE measurements are performed through intercostal spaces with the patient lying in the supine position with the left arm in maximal abduction. In the absence of guidelines, the same methods for liver stiffness measurements will be applied, performing one SWE measurement in the upper, mid and lower part of the spleen. A preliminary study on 30 subjects will be used to establish intraobserver and interobserver reproducibility.

Appendix 2:Standard operational procedures for Transient elastography of liver and spleen using FibroScan (Echosens, France)

1. The patient must be fasting form midnight, however a glass of water is allowed until 2 hours prior to the examination.
2. In the liver, TE measurements are performed on the right lobe, through intercostal spaces with the patient lying in the supine position with the right arm in maximal abduction. The same intercostal space should preferably be used for TE measurements and liver biopsy. The biopsy is done after TE.
3. Ten valid measurements should be obtained with a success rate of >60%
4. For the TE to be approved the IQR must be <30% of the median.

Statistical Analysis Plan

Version	2.1, dated 21/09/2021
Study title	Anti-fibrotic and molecular aspects of rifaximin in alcohol-related liver disease: A randomized placebo controlled clinical trial
Short title	The Rifaxmin study
Trial number	EudraCT: 2014-001856-51
Study protocol version	2.5, dated September 2019
Sponsors	Professor, Aleksander Krag, MD, PhD<br>Department of Medical Gastroenterology<br>Odense University Hospital<br>Sdr. Boulevard 29<br>5000 Odense C<br>Tlf: +4521409915<br>Mail: aleksander.krag@rsyd.dk
Principal Investigator	Mads Israelsen, MD, PhD<br>Department of Medical gastroenterology<br>Odense University Hospital<br>Sdr. Boulevard 29<br>5000 Odense C<br>Tlf:+45 51 82 99 58<br>Mail: mads.egerod.israelsen@rsyd.dk
Data manager responsible	M. Sc. Eng. Peter Andersen
SAP writer / contact person	MD, PhD, Mads Israelsen

Tool Revision History
Version Number: 1.0
Version Date (MM/YYYY): 2014

Version Number: 1.1
Version Date (MM/YYYY): September 2019
Summary of Revisions Made:

- Changes of principal investigator, updated recruitment strategi and replacement of the Ishak score fibrosis scoring system with the Kleiner fibrosis scoring system

Version Number: 2.0
Version Date (MM/YYYY): May 2021
Summary of Revisions Made:

- SAP from protocol implemented in the template of the SAP based on guidelines from Open Patient data Exploratory Network (OPEN)

Version Number: 2.1
Version Date (MM/YYYY): September 2021
Summary of Revisions Made:

- Addition of the statistical commands for the assessments the primary outcome and other binary outcomes, and definition of the mITT population.

1 LIST OF ABBREVIATIONS

ADS	Alcohol Dependency Scale
AE	Adverse events
ALD	Alcohol-related liver disease
ALP	Alkaline phosphatase
ALT	Alanine transaminase
AST	Aspartate aminotransferase
CLDQ	Chronic liver disease questionnaire
CRP	C-reactive protein
eCRF	Electronic case report format
FFQ	Food frequency questionnaire
GGT	Gamma-glutamyl transferase
HbA1c	Hemoglobin A1c
HDL	High-density lipoprotein
ITT	Intention-to-treat
LBP	Lipopolysaccharide binding protein
LDL	low-density lipoprotein
LSM	Liver stiffness measure
LPS	Lipopolysaccharide
MELD	Model for End-stage Liver Disease
mITT	Modified intention-to-treat
NAFLD	Non-alcoholic fatty liver disease
OR	Odds Ratio
PACS	Penn Alcohol Craving Scale
PI	Principal Investigator
PRO-C3	N-terminal pro-peptide of type III collagen
PRO-C4	Internal epitope in the 7S domain of type IV collagen
PRO-C8	C-terminal of type VIII collagen
PP	Per-protocol
SAE	Serious adverse events
SBP	Spontaneous bacterial peritonitis
SD	Standard deviation
SF-36	Short Form 36
SMT	Standard Medical Treatment
SNP	Single nucleotide polymorphism
TE	Transient elastography

2 STATISTICAL PRINCIPLES

2.1 Confidence intervals and P-values

A p-value < 0.05 will be considered statistically significant. P-values will be computed for both the primary and secondary outcomes regardless of the result of the primary outcome. 95% confidence intervals will be computed for all analyses.

2.2 Statistical Procedures

For baseline data we will report categorical data as counts and frequencies, and continuous data as means with standard deviations (SD) or medians with interquartile ranges (25%-75% percentile) according to distribution of data. All outcome data will be handled as paired data. For binary outcome on efficacy and harm of the intervention we will be assessed using logistics regression and will be reported as odds ratio (OR), its standard error and 95% confidence interval. Sensitivity analysis will be performed using logistic regression analysis adjusting for variables suspected to influence fibrogenesis such as age, sex, and alcohol consumption. Continuous outcome will be compared using paired t-test and Wilcoxon test according to the data distribution.

Before termination of the study and unblinding, Stine Johansen and Mads Israelsen have been writing the statistical commands for the assessments the primary outcome and other binary outcomes (see section 9 Statistic commands). Before termination of the study, the commands have been tested using randomly generated variables to simulate the outcomes.

2.3 Adherence and protocol deviations

During the course of the study, in situations where a deviation from the protocol is unavoidable, the study personnel in attendance will contact the appropriate co-investigator who will contact the principal investigator. Except in emergency situations, this contact should be made before implementing any departure from the protocol. In all cases, contact with the sponsor must be made as soon as possible to discuss the situation and agree on an appropriate course of action. The data recorded in the eCRF and source documents will reflect any departure from the protocol, and the source documents will describe this departure and the circumstances requiring it. A protocol deviation tracking log will be developed in order to track any non-adherence to the study protocol. Co-investigators and personnel will be responsible for updating the log with all relevant deviations. The deviations will be coded according to their deviation type and used for internal quality assurance. Any protocol deviation should also be reported to the local Independent Ethics Committee whenever applicable.

2.4 Analysis populations

Three populations will be defined in this study.

- The intention-to-treat (ITT) population is defined as all randomized patients.
- The modified intention-to-treat (mITT) population is defined as all randomized patients, who received at least one dose of the intervention.
- The per-protocol (PP) population is defined as all patients in the ITT population who did not present serious protocol violations, who ingested at least 75 % of the treatment and who was not withdrawn from the study due to non-adherence (interruption of treatment for four weeks or more). Efficacy and safety analyses will be performed on the PP and ITT population. Participants without an adequate liver biopsy at the end of the study cannot be

included in primary efficacy analysis of fibrosis score. Participants without an adequate liver biopsy participants will be included in the secondary endpoint analyses where we will use surrogate markers of liver fibrosis (transient elastography, Fibrosis-4 index, and PRO-C3 and PRO-C4). Sensitivity analyses on efficacy and safety will be performed in the PP and ITT populations.

3 STUDY DESIGN

Patients will be randomised 1:1 to receive rifaximin or placebo for 18 months. The study will be blinded, so neither patients nor medical staff will know whether the patient receives placebo or rifaximin.

4 TRIAL POPULATION

4.1 Recruitment

From 2012 an observational study at the department of medical gastroenterology evaluated the role of the microbiota and the development of hepatic fibrosis. The inclusion criteria for this study were: a) age 18-75 years and b) at least one year of alcohol abuse ≥24 grams/day for women and ≥36 grams/day for men. Exclusion criteria for this study were a) contraindications for liver biopsy, b) cancer or other debilitating disease with a life expectancy <1 year, c) concurrent liver disease other than alcohol, d) Human Immunodeficiency Virus, e) severe alcoholic hepatitis, f) not able to speak or read danish. Among these, a subset of 136 patients was planned to be included in this study. The enrolment of participants in this study has stopped and consequently a new recruitment strategy was initiated from October 2019 without changing the inclusion criteria. Accordingly, the inclusion criteria from the observational study is now integrated in the present study:

4.2 Inclusion criteria

5. Liver fibrosis score between 1 and 4 according to the Kleiner score

From the beginning of the trial in 2014 until 2019 the Ishak score[1] was used to stage liver fibrosis. From 2019 the stage of fibrosis was scored according to the Clinical Research Network staging system for NAFLD.[2] The reason for the revision was, that no accepted fibrosis grading system for ALD existed at the time of the study initiation. Since, European guidelines on the management of ALD propose NAFLD scoring systems as alternatives for fibrosis staging due to the large histological overlap between ALD and NAFLD.[3]

6. Age 18-75 years
7. At least one year of alcohol abuse ≥24 grams/day for women and ≥36 grams/day for men.
8. Women of child-bearing potential should use safe anti-conception and provide a negative pregnancy test.

Intra uterine device, and non-oral hormonal contraceptives (dermal, vaginal, implant or injection) will be considered safe during trial. According to SPC, oral hormone contraceptive will not be considered safe anti-conception. Instead use of double barrier, will be accepted. The terminal half – life for rifaximin is 4.17 hour so patient must continue with contraceptives minimum 24 hours after last dose.

4.3 Exclusion criteria

10. Known allergy to rifaximin
11. The investigator judge that the patient would not be compliant with trial medicine
12. Antibiotic treatment the prior 4 weeks
13. Contraindications for liver biopsy
14. Cancer or other debilitating disease with a life expectancy <1 year
15. Concurrent liver disease other than alcohol
16. Human Immunodeficiency Virus
17. Severe alcoholic hepatitis
18. Not able to speak or read Danish.
19.

4.4 Withdrawal from the study

5. Blinding is repealed
6. Treatment with another antibiotic for more than 4 consecutive weeks or 4 times during the study
7. The trial participant withdraws his/her written consent
8. It is considered in the participants' best interest, as judged by investigator

4.5 Dropout

Trial participants will be classified as dropouts if they meet any of the following criteria

3. The trial participant has ingested less than 75 % planned treatment
4. The trial participants do not attend planned controls, despite contact by telephone, letter or mail

If possible, the following data on dropouts will be registered: Reason for dropout, mortality, amount of ingested trial medicine, all patient data collected during the trial.

4.6 Recruitment and enrolment of participants

Physician investigators associated to ongoing research projects on alcohol-related liver disease at Liver Research Centre will identify patients eligible for trial. Patients will receive oral information about the trial and be offered inclusion. Trial information and written consent forms is handed out to the patient. Subsequently the patient will sign the informed consent if they accept to participate in the trial.

4.7 Time schedule

1/9-2014 Enrolment of first patient and start of investigations
31/07-2020 Enrolment of last patient
31/12-2021 End of follow up, trial shut-down
30/06-2022 First manuscript on the manuscript on the efficacy and safety of rifaximin
31/12-2031 End of analysis and data assessment

4.8 Screening data

Everyone evaluated for inclusion will be entered into a screening log. When patients are not eligible to participate in the study, a reason for exclusion will be entered into the screening log. Any patient who retract their informed consent prior to randomization will be categorized as ‘refused to participate’.

5 ANALYSES

5.1 Baseline patient characteristics

Variables with reporting methods and unit of measurement [unit]. Reporting of continuous variables depends on the distribution. For normally distributed variables, mean and standard deviations (SD) are reported. Non-normally distributed variables are reported as medians and interquartile ranges. Checks of normality for baseline characteristics will be considered in a Shapiro-Wilk test and a QQ-plot.

Demographic data
- Age (mean, SD, [years])
- Gender (N, % male)
- Ethnicity (N, % of total)

Clinical features
- Height (mean, SD, [cm])
- Weight (mean, SD, [kg])
- Handgrip strength (mean, SD, [kg])
- Body-mass index (mean, SD, [kg/m^2])
- Systolic arterial pressure (mean, SD, [mmHg])
- Diastolic arterial pressure (mean, SD, [mmHg])
- Mean arterial pressure (mean, SD, [mmHg])
- Heart rate (mean, SD, beats per minute)
- LSM by TE (mean, SD, [kPa])

Laboratory data
- White blood cells (mean, SD, [10^9 cells per L])
- Haemoglobin (mean, SD, [g/dL])
- Platelets (mean, SD, [10^9 cells per L])
- Serum creatinine (mean, SD, [mg/dL])
- Urea (mean, SD, [mmol/L])
- Serum sodium (mean, SD, [mmol/L])
- Serum potassium (mean, SD, [mmol/L])
- Serum bilirubin (mean, SD, [mg/dL])
- Serum albumin (mean, SD, [g/L])
- ALT (mean, SD, [U/L])
- AST (mean, SD, [U/L])
- GGT (mean, SD, [U/L])
- ALP (mean, SD, [U/L])
- Fibrosis-4 index
- International normalised ratio, INR (mean, SD, [ratio])
- Fasting plasma glucose (mean, SD, [mmol/L])
- HbA1c (mean, SD, [mmol/mol])
- Triglycerids (mean, SD, [mmol/L])
- Total cholesterol (mean, SD, [mmol/L])
- LDL (mean, SD, [mmol/L])
- HDL (mean, SD, [mmol/L])
- CRP (mean, SD, [mg/L])

Genetics

- Risk single nucleotide polymorphisms (SNPs) including PNPLA3, TM6SF2, MBOAT7, HSD13B17

Medication

- Antidiabetic drugs (active substance group, % of total)
- Diuretic drugs (active substance group, % of total)
- Anti-hypertensive drugs (active substance group, % of total)
- Proton pump inhibitors (N, % of total)
- H_2 inhibitors (N, % of total)
- Anti-depressive drugs (active substance group, % of total)
- Anxiolytics (active substance group, % of total)
- Pain killers (active substance group, % of total)

Clinical history

- Comorbidities (N, % of total)
- Cardiovascular disease (N, % of total)
- Pulmonary disease (N, % of total)
- Gastro-intestinal disease (N, % of total)
- Endocrinological disorder (N, % of total)
- Genito-urinary disease (N, % of total)
- Musculoskeletal disorders (N, % of total)

Quality of life

- CLDQ (mean, SD)
- SF-36 (mean, SD)

Patterns of alcohol consumption

- Penn Alcohol Craving Scale (PACS)
- Alcohol Dependency Scale (ADS)

Diet

- FFQ (mean, SD)
- 24h Dietary Recall (mean, SD)

5.2 Outcome measurement

5.2.1 Primary outcome

All histological assessments will be rescored at the end of the trial. Outcome assessment will we performed by one pathologist (Sönke Detlefsen) with expertise in liver histology and blinded to the clinical data. Liver biopsies will be considered of adequate quality if they are of >10 mm length and >6 portal tracts or presence of cirrhotic nodules.

The primary outcome will be a between-treatment group comparison of the proportion of participants with an improvement in liver fibrosis score equal to or more than 1.

In addition, we will perform a between-treatment group comparison of the proportion of participants with a progression in liver fibrosis score equal to or more than 1.

To assess liver fibrosis, we will use the fibrosis staging system developed by the Clinical Research Network staging system for NAFLD[2]. This is an adjustment of the original protocol[4], where we planned to use Ishak score for chronic hepatitis[1]. The reason for the revision was, that no accepted fibrosis grading system for ALD existed at the time of the study initiation. Since, European guidelines on the management of ALD propose NAFLD scoring systems as alternatives for fibrosis staging due to the large histological overlap between ALD and NAFLD.[3]

5.2.2 Secondary outcomes

A. Markers of fibrosis

Histological: collagen proportionate area
Ultrasound: liver stiffness assessed by transient elastography
Fibrosis-4 index
Plasma/serum: Enhanced liver fibrosis (ELF) test, PRO-C3, PRO-C4, PRO-C8

B. Markers of inflammation

Histological: improvement of lobular inflammation, hepatocyte ballooning according to the Clinical Research Network staging system for NAFLD.[2]

Plasma/Serum: Proinflammatory cytokines: tumor necrosis factor α, monocyte chemoattractant protein 1, and cluster of differentiation 163.

C. Markers of matrix remodeling

Definition: tissue inhibitor of metalloproteinase 1, matrix metallopeptidase 2, and profibrotic cytokines (transforming growth factor β1, platelet-derived growth factor β, and connective tissue growth factor)

D. Composition of the gut microbiota

Definition: 16S and shotgun metagenomics sequencing of faeces.

E. Markers of gut integrity and leaky gut

Definition: lipopolysaccharide (LPS), lipopolysaccharide binding protein (LBP), CPa9-HNE

F. Standard laboratory data
White blood cells (mean, SD, [10^9 cells per L]), Haemoglobin (mean, SD, [g/dL]), Platelets (mean, SD, [10^9 cells per L]), Creatinine (mean, SD, [mg/dL]), Urea (mean, SD, [mmol/L]), Sodium (mean, SD, [mmol/L]), Potassium (mean, SD, [mmol/L]), Bilirubin (mean, SD, [mg/dL]), Albumin (mean, SD, [g/L]), ALT (mean, SD, [U/L]), AST (mean, SD, [U/L]), GGT (mean, SD, [U/L]), ALP (mean, SD, [U/L]), International normalised ratio, INR (mean, SD, [ratio]), Fasting plasma glucose (mean, SD, [mmol/L]), HbA1c (mean, SD, [mmol/mol]), Triglycerids (mean, SD, [mmol/L]), Total cholesterol (mean, SD, [mmol/L]), LDL (mean, SD, [mmol/L]), HDL (mean, SD, [mmol/L]), CRP (mean, SD, [mg/L]), Alpha-foetoprotein (mean, SD, [10^3 IU/L])

G. Quality of life
Questionnaires: Short Form (36) Health Survey and Chronic Liver Disease Questionnaire

H. Nutritional status
Body weight and hand grip strength

I. Alcohol consumption

- Penn Alcohol Craving Scale (PACS)
- Alcohol Dependency Scale (ADS)

J. Adverse events

Adverse events (AE) and serious adverse events (SAE) are defined according to the Guideline for Good Clinical Practice E6(R2). The adverse events are classified according to their attribution status and severity criteria.

Attribution definition	Severity criteria
**Not Related**: An AE that is not related to the use of the drug.<br>**Doubtful**: An AE for which an alternative explanation is more likely, eg, concomitant drug(s), concomitant disease(s), or the relationship in time suggests that a causal relationship is unlikely.<br>**Possible**: An AE that might be due to the use of the drug. An alternative explanation, eg, concomitant drug(s), concomitant disease(s), is inconclusive. The relationship in time is reasonable; therefore, the causal relationship cannot be excluded.<br>**Probable**: An AE that might be due to the use of the drug. The relationship in time is suggestive (eg, confirmed by dechallenge). An	**Mild (GRADE 1)**: Transient or mild discomfort (< 48 hours); no medical intervention/therapy required<br>**Moderate (GRADE 2)**: Mild to moderate limitation in activity - some assistance may be needed; no or minimal medical intervention/therapy required<br>**Severe (GRADE 3)**: Marked limitation in activity, some assistance usually required; medical intervention/therapy required, hospitalizations possible<br>**Life-threatening (GRADE 4)**: Extreme limitation in activity, significant assistance required; significant medical intervention/therapy required, hospitalization or hospice care probable

alternative explanation is less likely, eg, concomitant drug(s), concomitant disease(s).<br>**Very Likely**: An AE that is listed as a possible adverse reaction and cannot be reasonably explained by an alternative explanation, eg, concomitant drug(s), concomitant disease(s). The relationship in time is very suggestive (eg, it is confirmed by dechallenge and rechallenge).	

Number of adverse events during treatment
Definition: defined as any untoward medical occurrence in a patient participating in the trial. An Adverse event (AE) does not necessarily have a causal relationship with treatment. An adverse event can therefore be an unintended sign (including an abnormal finding), symptom, or disease temporally associated with the treatment, whether or not it is related to infusion of treatment. This includes worsening of a pre-existing condition or increase in frequency of a pre-existing condition. Source of information: eCRF

Number of serious adverse events
Definition: defined as any untoward medical occurrence happening to the trial participants that: Results in death, is life-threatening, results in persistent or significant disability/incapacity, is a congenital abnormally or is a suspected transmission of any infectious agent via a medicinal product. Apart from this, any event that requires hospitalization or prolongs an existing hospitalization during the cause of a patient's participation in the study must be reported as an SAE. Other medical events that are not included in the above definitions can be reported as an SAE by the treating physician if he or she considers it appropriate to do so. A SAE does not necessarily have a causal relationship with the treatment

5.3 Subgroup analyses

We plan to perform subgroup analyses according to the following:

- Ongoing alcohol use at inclusion
- Alcohol use during the intervention
- Genotype
- Gut dysbiosis

5.3 Sensitivity analyses

We plan to perform sensitivity analyses according to the following:

- Gender
- Age
- alcohol abstinence six months prior to inclusion

5.4 Missing data

Any missing values will not be imputed after the termination of the study. The statistical analysis will be performed in an available data only fashion. However, to allow for the mITT analyses for the histological outcomes (including the primary outcome), participants receiving at least one dose of the intervention will be included in the mITT analyses and participants with missing data will be considered as having had no response (stable) in line with a previous study investigating the efficacy of semaglutid on liver histological in patients with non-alcoholic steatohepatitis.[7]

5.5 Statistical Software

All calculations will be performed in STATA (College Station, TX, US), R (R Foundation for Statistical Computing, Vienna, Austria) and SAS (SAS Institute).

6 REFERENCES

1. Ishak K, Baptista A, Bianchi L, et al. Histological grading and staging of chronic hepatitis. *J Hepatol*. Jun 1995;22(6):696-9. doi:10.1016/0168-8278(95)80226-6
2. Kleiner DE, Brunt EM, Van Natta M, et al. Design and validation of a histological scoring system for nonalcoholic fatty liver disease. *Hepatology*. Jun 2005;41(6):1313-21. doi:10.1002/hep.20701
3. European Association for the Study of the Liver. Electronic address eee, European Association for the Study of the L. EASL Clinical Practice Guidelines: Management of alcohol-related liver disease. *J Hepatol*. Jul 2018;69(1):154-181. doi:10.1016/j.jhep.2018.03.018
4. Madsen BS, Trebicka J, Thiele M, et al. Antifibrotic and molecular aspects of rifaximin in alcoholic liver disease: study protocol for a randomized controlled trial. *Trials*. Feb 26 2018;19(1):143. doi:10.1186/s13063-018-2523-9
5. Israelsen M, Guerrero Misas M, Koutsoumourakis A, et al. Collagen proportionate area predicts clinical outcomes in patients with alcohol-related liver disease. *Aliment Pharmacol Ther*. Oct 12 2020;doi:10.1111/apt.16111
6. Lackner C, Stauber RE, Davies S, et al. Development and prognostic relevance of a histologic grading and staging system for alcohol-related liver disease. *J Hepatol*. Jun 11 2021;doi:10.1016/j.jhep.2021.05.029
7. Newsome PN, Buchholtz K, Cusi K, et al. A Placebo-Controlled Trial of Subcutaneous Semaglutide in Nonalcoholic Steatohepatitis. *N Engl J Med*. Mar 25 2021;384(12):1113-1124. doi:10.1056/NEJMoa2028395

7 FIGURES AND TABLES

CONSORT

TRANSPARENT REPORTING of TRIALS

CONSORT 2010 Flow Diagram

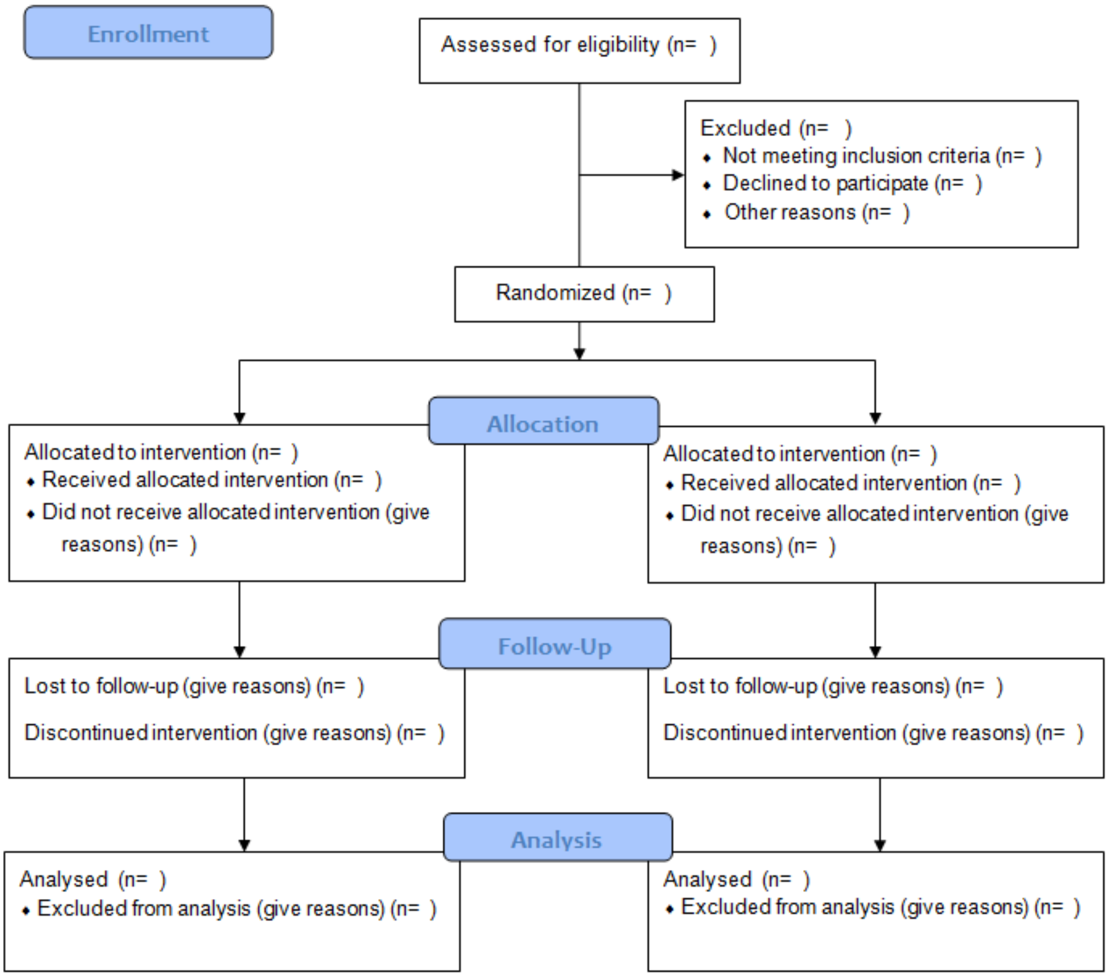

Figure 1 –CONSORT flow diagram

8 Statistical commands

Commands to be used in Stata for 1) between-treatment group comparison of the proportion of participants with an improvement in liver fibrosis score equal to or more than 1 and 2) a between-treatment group comparison of the proportion of participants with a progression in liver fibrosis score equal to or more than 1.

```
use "GALA-RIF", clear

keep rfx_id gruppe visit CRNFscore
reshape wide CRNFscore, i(rfx_id gruppe) j(visit)
gen fib_dif=CRNFscore_after-CRNFscore_before

*** Used only for the PP-analysis ***
drop if fib_dif==.

*** Used only for the mITT ***
replace fib_outcome=2 if fib_dif==.
drop if mITT==0

gen fib_outcome=.
replace fib_outcome=1 if fib_dif<0
replace fib_outcome=2 if fib_dif==0
replace fib_outcome=3 if fib_dif>0

label define fib_outcome_label 1 "Regression" 2 "Stable" 3 "Progression"
label values fib_outcome fib_outcome_label

tab fib_outcome gruppe, col chi2

graph bar, over(gruppe) over(fib_outcome) graphregion(color(white)) bgcolor(white)
ylabel(0(10)40) title("Fibrosis", size(huge))

*** improvement vs no improvement
gen improvement=0
replace improvement=1 if fib_outcome==1
tab improvement gruppe, row chi2
logit improvement gruppe, or

*** worsening vs no worsening
gen worsening=1
replace worsening=0 if fib_outcome==3
tab worsening gruppe, col chi2
logit worsening gruppe, or
```
